# The Nr4a family regulates intrahepatic Treg proliferation and liver fibrosis in MASLD models

**DOI:** 10.1172/JCI175305

**Published:** 2024-10-15

**Authors:** Daisuke Aki, Taeko Hayakawa, Tanakorn Srirat, Shigeyuki Shichino, Minako Ito, Shin-Ichiroh Saitoh, Setsuko Mise-Omata, Akihiko Yoshimura

**Affiliations:** 1 Department of Microbiology and Immunology, Keio University School of Medicine, Tokyo, Japan.; 2 Department of Intractable Disorders, Institute of Advanced Medicine, Wakayama Medical University, Wakayama, Japan.; 3 Division of Molecular Regulation of Inflammatory and Immune Diseases, Research Institute for Biomedical Sciences, Tokyo University of Science, Chiba, Japan.; 4 Division of Allergy and Immunology, Medical Institute of Bioregulation, Kyushu University, Fukuoka, Japan.; 5 Division of Molecular Pathology, Research Institute for Biomedical Science, Tokyo University of Science, Chiba, Japan.

**Keywords:** Immunology, Fibrosis, T cell development, T cells

## Abstract

Metabolic dysfunction–associated steatotic hepatitis (MASH) is a chronic progressive liver disease that is highly prevalent worldwide. MASH is characterized by hepatic steatosis, inflammation, fibrosis, and liver damage, which eventually result in liver dysfunction due to cirrhosis or hepatocellular carcinoma. However, the cellular and molecular mechanisms underlying MASH progression remain largely unknown. Here, we found an increase of the Nr4a family of orphan nuclear receptor expression in intrahepatic T cells from mice with diet-induced MASH. Loss of Nr4a1 and Nr4a2 in T cell (dKO) ameliorated liver cell death and fibrosis, thereby mitigating liver dysfunction in MASH mice. dKO resulted in reduction of infiltrated macrophages and Th1/Th17 cells, whereas it led to a massive accumulation of Tregs in the liver of MASH mice. Combined single-cell RNA transcriptomic and TCR sequencing analysis revealed that intrahepatic dKO Tregs exhibited enhanced T cell immunoreceptor with Ig and ITIM domains (TIGIT) and IL-10 expression and were clonally expanded during MASH progression. Mechanistically, we found that dKO Tregs expressed high levels of basic leucine zipper ATF-like transcription factor (Batf), which promotes Treg cell proliferation and function upon TCR stimulation. Collectively, our findings not only provide an insight into the impact of intrahepatic Treg cells on MASH pathogenesis, but also suggest a therapeutic potential of targeting of the Nr4a family to treat the disease.

## Abstract 2

Nr4a family regulates Tregs proliferation and function in the liver during NASH progression.

## Introduction

Metabolic dysfunction-associated steatotic liver disease (MASLD) is a liver disorder characterized by the accumulation of fat in the liver without significant alcoholic intake, and its prevalence is estimated to be around 25% (1). MASLD includes a range of liver conditions from simple steatosis to a more aggressive form of MASLD, known as metabolic dysfunction–associated steatotic hepatitis (MASH). MASH occurs in approximately 20% of patients with MASLD and is characterized by steatosis, persistent inflammation, liver damage, and fibrosis (2, 3). MASH increases the risk for liver cirrhosis and hepatocellular carcinoma (HCC) and is also associated with prevalence and incidence of cardiovascular disease, chronic kidney disease, and type 2 diabetes (1, 4). Despite intense clinical trials, currently there are no effective therapies for MASH (5), since the cellular and molecular mechanisms underlying MASH progression are not fully understood.

The liver contains diverse innate and adaptive immune cells within its tissue microenvironment. Innate immune response has been considered critical for MASH development, acting by triggering and propagating liver inflammation (6). Single-cell RNA sequencing (scRNA-Seq) studies have revealed the transcriptomic landscape of hepatic macrophages and identified the key molecules associated with MASH pathology (7–9). However, it has also recently emerged that T lymphocyte–mediated adaptive immunity regulates liver homeostasis in MASH (10, 11). For instance, MASH leads to an accumulation of CXCR6^+^ CD8 T cells, which promote MASH pathology and development of MASH-induced HCC (12, 13). MASLD causes a selective loss of hepatic CD4^+^ T cells, resulting in accelerated hepatocarcinogenesis (14). Meanwhile, the roles of CD4^+^ T helper cell subsets, such as IFN-γ–producing Th1 and IL-17–producing Th17, have also been shown to be involved in MASH (10). Tregs, which are indispensable for immune system homeostasis, acting by suppressing effector T cell response, were decreased in MASLD mice, and adaptive transfer of Tregs attenuated liver inflammation when the mice were challenged by lipopolysaccharide (15). Another study demonstrated that Tregs were selectively increased and depletion of Tregs led to inhibition of HCC initiation (16). Conversely, transfer of Tregs into MASH-induced BALB/c *Rag1^–/–^* mice worsened liver inflammation (17). Thus, the current understanding of how Tregs affect MASH pathology remains elusive. Additionally, although transcriptionally and functionally distinct Treg subpopulations from these counterparts in lymphoid organ, known as tissue Tregs, have been identified (18), it is largely unknown whether such cells could control MASH development.

The Nr4a family comprises Nr4a1, Nr4a2, and Nr4a3, which are nuclear orphan receptor–type transcription factors. We previously reported that the Nr4a family is essential for the induction of Tregs in the thymus and also involved in the maintenance of Tregs in the periphery (19, 20). Interestingly, hepatic mRNA expression of Nr4a was shown to be elevated in diabetic mice, and adenoviral expression of Nr4a1 increased blood glucose levels via promoting gluconeogenesis (21).

Given the aforementioned studies, we aimed to elucidate the impact of hepatic Tregs in MASH and whether Nr4a family regulates T cell–mediated tissue homeostasis during MASH. In this study, we found an upregulation of Nr4a family expression in hepatic T cells during MASH progression. Mice lacking all *Nr4a* (*Nr4a1*, *Nr4a2*, and *Nr4a3*) showed severe reduction of thymic and peripheral Tregs (20). However, unexpectedly, specific loss of *Nr4a1* and *Nr4a2* in T cells in mice (dKO mice) ameliorated MASH pathology and Tregs were accumulated in the liver. Integrated transcriptome and TCR clonotype data revealed that hepatic Tregs lacking *Nr4a1* and *Nr4a2* exhibited strongly immunosuppressive phenotypes and underwent clonal expansion during MASH, accompanied with strong upregulation of *Nr4a3*. Furthermore, we identified target gene basic leucine zipper ATF-like transcription factor (*Batf*), whose expression was increased in *Nr4a1*- and *Nr4a2*-deficient Tregs and found that it regulated Treg proliferation and function. Batf has been shown to play an important role in Treg residency in nonlymphoid tissues (22). Together, our findings suggest the protective function of hepatic Tregs during liver inflammation and highlight the role of the Nr4a-Batf circuit in regulating Treg expansion.

## Results

### Expression of the Nr4a family is upregulated in hepatic T cells from MASH mice.

**Figure 1:**
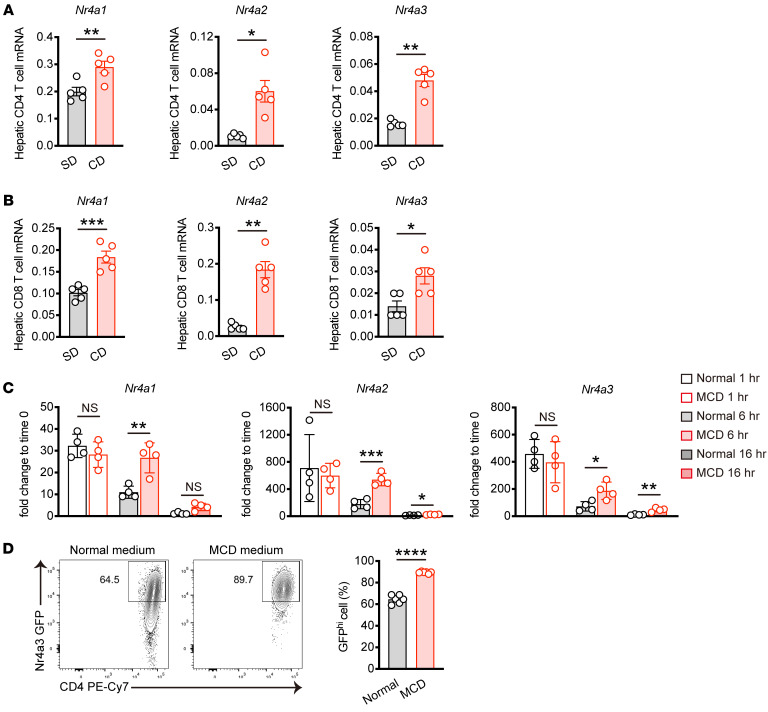
MASH upregulates Nr4a family expression in hepatic T cells. (**A** and **B**) Male C57BL/6 mice were fed SD or CD for 12 weeks (*n* = 5 per group). mRNA expression of *Nr4a1*, *Nr4a2*, and *Nr4a3* in hepatic CD4^+^ T cells (**A**) or hepatic CD8^+^ T cells (**B**). (**C**) mRNA expression of *Nr4a1*, *Nr4a2*, and *Nr4a3* in stimulated CD4^+^ T cells. Splenic naive CD4^+^ T cells were stimulated with α-CD3 and α-CD28 antibodies in normal RPMI (normal), or methionine and choline-deficient (MCD) medium for the indicated times (*n* = 4 per group). (**D**) Splenic naive CD4^+^ T cells from Nr4a3-EGFP reporter mice were stimulated with α-CD3 and α-CD28 antibodies in normal RPMI or MCD medium for 3 days, and GFP expression was assessed by flow cytometry. Representative flow cytometry plots (left) and percentages (right) of GFP^hi^ cells (*n* = 6 per group). Primers used for quantitative PCR are listed in Supplemental Table 1. Data are represented as means ± SEM. *P* values were calculated using unpaired 2-tailed Student’s *t* test or Mann-Whitney *U* test (**A**–**D**). *P < 0.05; **P < 0.01; ***P < 0.001; ****P < 0.0001.

To investigate the function of the Nr4a family in T cells during MASH progression, we subjected mice to a choline-deficient, high-fat diet consisting of 60 kcal% fat and 0.1% methionine (CD) for 8 weeks (23). After 8 weeks of CD feeding, we observed liver steatosis and fibrosis, which are characteristics of MASH (Supplemental Figure 1, A–C; supplemental material available online with this article; https://doi.org/10.1172/JCI175305DS1). Subsequently, we investigated the mRNA expression levels of the *Nr4a* family in hepatic T cells from mice fed CD for 12 weeks to further progress MASH development. Gene expression of *Nr4a1*, *Nr4a2*, and *Nr4a3* was significantly upregulated in hepatic CD4^+^ and CD8^+^ T cells from MASH mice (Figure 1, A and B). To further determine whether MASH induces Nr4a family expression, we cultured naive CD4^+^ T cells with substitution of RPMI medium deficient in methionine and choline (MCD medium) mimicking the in vivo CD-induced MASH model, and analyzed the kinetics of *Nr4a* family gene expression. In normal RPMI medium, *Nr4a* family transcription was rapidly upregulated within 1 hour, and declined by 6 hours after stimulation, as previously described (19). While no significant difference in the induction of *Nr4a* family mRNA was observed between CD4^+^ T cells cultured in normal RPMI and MCD medium at 1 hour, sustained transcription of the Nr4a family was evident in CD4^+^ T cells cultured in MCD medium, persisting even at 16 hours after stimulation (Figure 1C). Finally, we directly assessed whether protein levels of the Nr4a family were increased in MCD medium. We isolated naive CD4^+^ T cells from transgenic mice expressing GFP from the *Nr4a3* locus and cultured these cells for 3 days in either normal or MCD medium. As a result, we found higher Nr4a3 expression levels in CD4^+^ T cells cultured in MCD medium compared with normal RPMI (Figure 1D). Altogether, these data show that the Nr4a family is upregulated in T cells during MASH progression.

### T cell–specific loss of Nr4a1 and Nr4a2 alleviates MASH development in mice.

**Figure 2:**
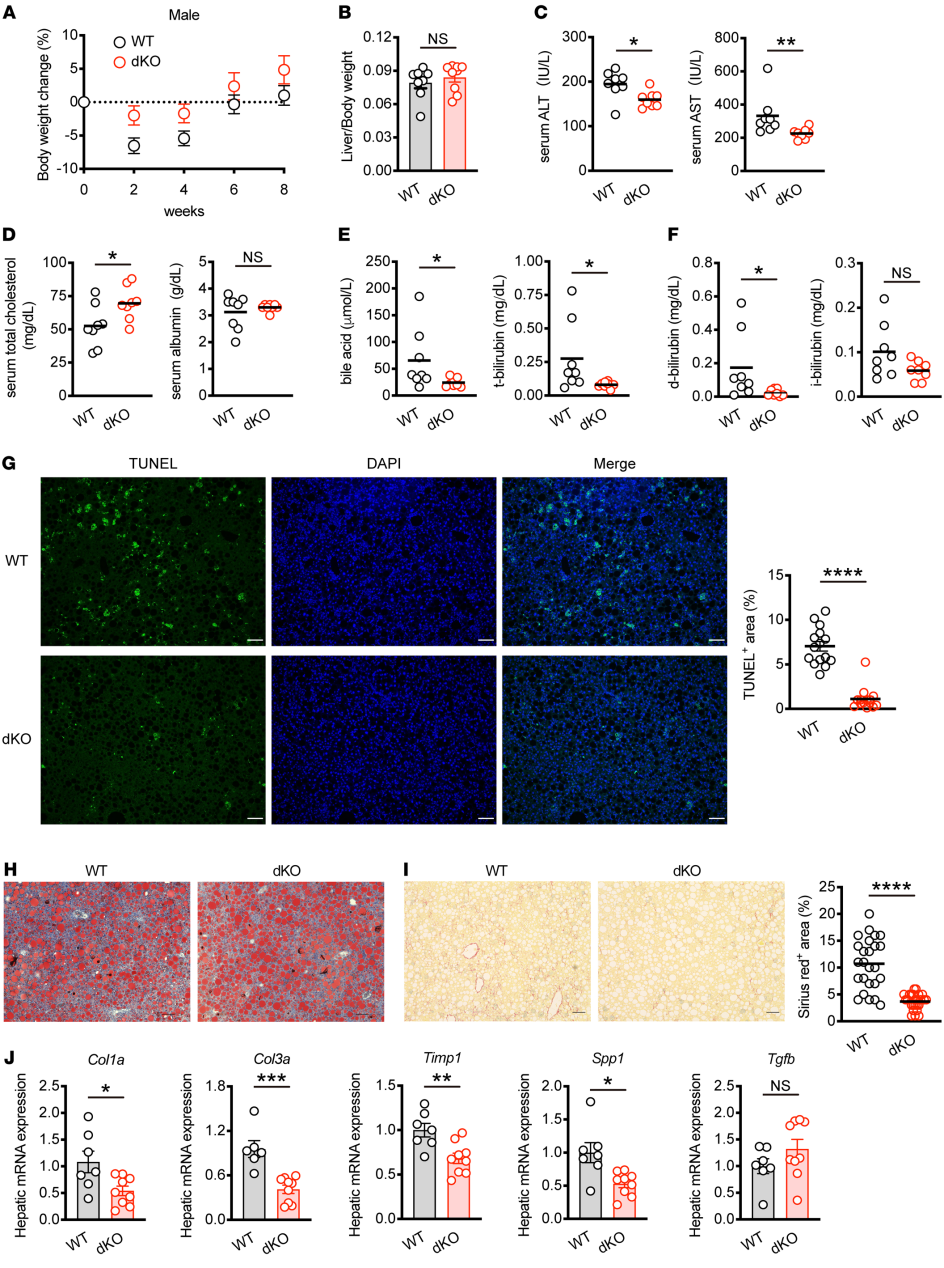
Loss of *Nr4a1* and *Nr4a2* in T cell alleviates MASH pathology. (**A**) Relative body weight changes of male WT and dKO mice fed CD for the indicated times (WT; *n* = 13, dKO; *n* = 14). (**B**) The ratio of liver weight to body weight of male WT and dKO mice fed CD for 8 weeks (WT; *n* = 8, dKO; *n* = 9). (**C**–**F**) Female WT and dKO mice were fed CD for 12 weeks (*n* = 8 per group). ALT and AST levels (**C**), total cholesterol, albumin levels (**D**), bile acid, total bilirubin (t-bilirubin) levels (**E**), direct bilirubin (d-bilirubin), indirect bilirubin (i-bilirubin) levels (**F**) in the serum. (**G**–**J**) Male WT and dKO mice were fed CD for 8 weeks. (**G**) Representative liver sections stained TUNEL (green) and DAPI (blue). Original magnification, ×10. Scale bars: 100 μm (left). Quantification of TUNEL-positive area (%) per field (right) (*n* = 3 per group). (**H**) Representative oil red O staining of liver sections from 2 independent experiments. Original magnification, ×10. Scale bars: 100 μm. (**I**) Representative Sirius red staining of liver sections. Original magnification, ×10. Scale bars: 100 μm (left). Quantification of Sirius red staining area (%) per field (right) (*n* = 5 per group). (**J**) mRNA expression of fibrosis-related genes in liver tissue (WT; *n* = 6–7, dKO; *n* = 9). Data are represented as means ± SEM. *P* values were calculated using unpaired 2-tailed Student’s *t* test or Mann-Whitney *U* test (**B**–**G**, **I**, and **J**). *P < 0.05; **P < 0.01; ***P < 0.001; ****P < 0.0001.

Given the significant upregulation of the Nr4a family in T cells with MASH, we postulated that the Nr4a family in T cells might impact MASH pathogenesis. To address this, we generated T cell–specific *Nr4a1*- and *Nr4a2*-deficient mice (*Nr4a1^fl/fl^Nr4a2^fl/fl^Cd4Cre* mice, referred to hereafter as dKO mice). Unlike mice lacking *Nr4a1*, *Nr4a2*, and *Nr4a3* (20), dKO mice exhibited normal percentages of naive and effector/memory CD4^+^ and CD8^+^ T cells in the spleen at steady state, suggesting restored T cell homeostasis in dKO mice (Supplemental Figure 2, A and B). Then WT and dKO mice were fed CD for 8 weeks to induce MASH. Body weight changes and liver–to–body weight ratios were similar between WT and dKO mice (Figure 2, A and B, and Supplemental Figure 2C). However, dKO mice exhibited lower levels of alanine aminotransferase (ALT) and aspartate aminotransferase (AST) in the serum compared with WT mice (Figure 2C), indicating that attenuated liver damage was caused by steatohepatitis in dKO mice. Consistently, dKO mice showed elevated levels of serum total cholesterol that include both HDL and LDL cholesterol, although albumin levels remained unchanged (Figure 2D). Furthermore, serum bile acid and bilirubin levels were lower in dKO mice, with a notable reduction in direct bilirubin, implying restricted bile duct obstruction during MASH progression (Figure 2, E and F). TUNEL staining demonstrated decreased liver cell death in dKO mice (Figure 2G). These results suggest that Nr4a1/2 in T cells promotes liver damage, leading to impaired liver function with MASH. However, lipid deposition in the liver, as shown by oil red O staining, was comparable between WT and dKO mice on CD (Figure 2H). Both groups of mice showed similar expression levels of lipid metabolism–related genes such as *Srebf1* (fatty acid biosynthesis) and *Ldlr* (lipid uptake) in the liver (Supplemental Figure 2D). However, liver fibrosis as assessed by Sirius red or Masson-trichrome staining was reduced in dKO mice (Figure 2I and Supplemental Figure 2E). α-Smooth muscle actin (α-SMA) expression was also downregulated in dKO mice (Supplemental Figure 2F). In line with the attenuated fibrosis, the expression of fibrosis marker genes was downregulated in the liver of dKO mice (Figure 2J). Collectively, these data suggest that the Nr4a family in hepatic T cells contributes to MASH development, partly through the promotion of liver fibrosis.

### dKO reduces infiltrated macrophages in the liver.

**Figure 3:**
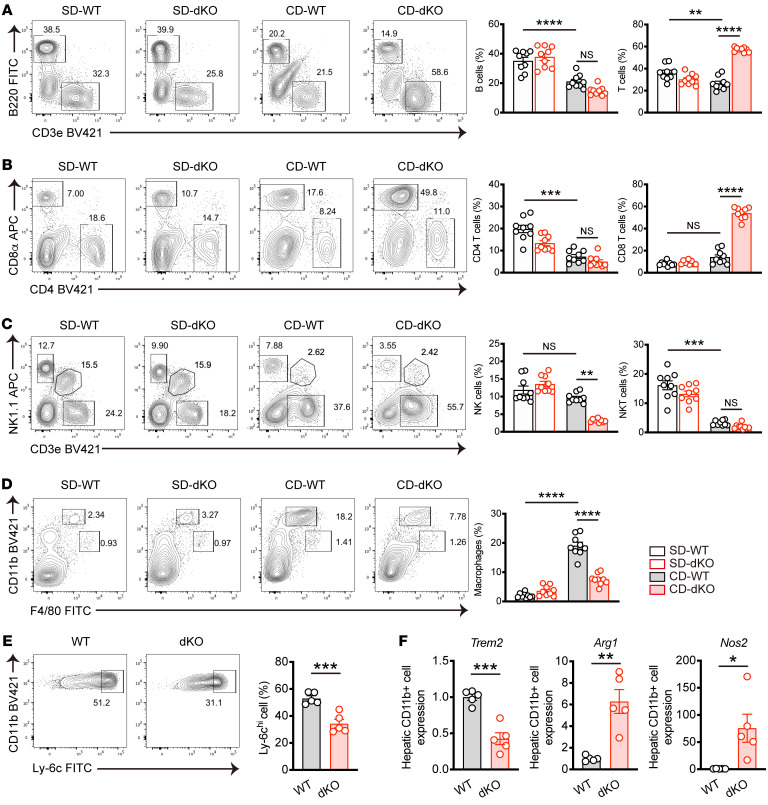
Hepatic immune cell composition from WT or dKO mice fed CD for 8 weeks. (**A**–**D**) WT and dKO mice were fed SD or CD for 8 weeks. (**A**) Representative flow cytometry plots (left) and percentages (right) of B cells (CD45^+^B220^+^) and T cells (CD45^+^CD3e^+^) in the liver (*n* = 9 per group). (**B**) Representative flow cytometry plots (top) and percentages (bottom) of CD45^+^CD4^+^ T cells and CD45^+^CD8 T cells in the liver (*n* = 9 per group). (**C**) Representative flow cytometry plots (left) and percentages (right) of NK (CD45^+^CD3e^–^NK1.1^+^) cells and NKT (CD45^+^CD3e^+^NK1.1^+^) cells in the liver (*n* = 9 per group). (**D**) Representative flow cytometry plots (left) of macrophages (CD45^+^CD11b^hi^F4/80^int^) and Kupffer cells (CD45^+^CD11b^int^F4/80^hi^) and percentages (right) of macrophages in the liver (*n* = 9 per group). (**E**) Representative flow cytometry plots (top) and percentages (bottom) of CD45^+^CD11b^hi^F4/80^int^Ly-6C^hi^ macrophages in the liver from WT and dKO mice fed CD for 8 weeks (*n* = 5 per group). (**F**) mRNA expression of *Trem2*, *Arg1*, and *Nos2* in hepatic CD11b^+^ cells from WT and dKO mice fed CD for 8 weeks (*n* = 5 per group). Data are represented as means ± SEM. *P* values were calculated using 1-way ANOVA or Kruskal-Wallis test (**A**–**D**) or unpaired 2-tailed Student’s *t* test (**E** and **F**). *P < 0.05; **P < 0.01; ***P < 0.001; ****P < 0.0001.

To explore the cellular mechanisms by which Nr4a1/2 loss in T cells alleviates MASH, we performed flow cytometry analysis of intrahepatic immune cell populations (Supplemental Figure 3A). Frequencies of B cell and T cell populations were reduced in WT mice on CD compared with standard diet (SD), whereas T cell populations were elevated in dKO mice upon MASH induction (Figure 3A and Supplemental Figure 3, B and C). Notably, increased T cells in dKO mice were primarily due to expansion of CD8^+^ T cells, but not CD4^+^ T cells (Figure 3B and Supplemental Figure 3, D and E). In addition, the percentages of hepatic CD3^–^NK1.1^+^ NK cells were reduced in MASH dKO mice, while CD3^int^NK1.1^+^ NKT cells remained comparable between WT and dKO mice fed CD (Figure 3C and Supplemental Figure 3, F and G). Hepatic macrophages are shown to play an important role in chronic liver inflammation and fibrosis (24). We therefore examined the infiltrated macrophages (CD11b^hi^F4/80^int^) and liver-resident Kupffer cells (CD11b^int^F4/80^hi^). In response to CD, WT mice showed greater expansion of infiltrated macrophages, indicative of hepatic inflammation. While there was no significant difference between WT and dKO mice fed SD, frequency of infiltrated macrophages was reduced in dKO mice under MASH conditions (Figure 3D and Supplemental Figure 3H). The Kupffer cell population in dKO mice was on the same level with that of WT mice regardless of the presence or absence of MASH (Figure 3D). Further analysis demonstrated that inflammatory macrophages defined as CD11b^hi^F4/80^int^Ly-6c^hi^ were significantly decreased in dKO mice (Figure 3E). Then we isolated CD11b^+^ cells from the liver and assessed marker gene expression that is related to pro- or antiinflammatory macrophages. *Trem2*, which is expressed on MASH-associated inflammatory macrophages (25,) was downregulated in macrophages from dKO mice. On the other hand, both *Arg1* and *Nos2* expression were augmented in macrophages from dKO mice (Figure 3F), suggesting an immunosuppressive rather than inflammatory phenotype (26–28). Collectively, these data demonstrate that loss of the Nr4a family in T cells leads to increased CD8^+^ T cells and reduction of infiltrated inflammatory macrophages during MASH.

### dKO mice contain more intrahepatic Tregs in MASH.

**Figure 4:**
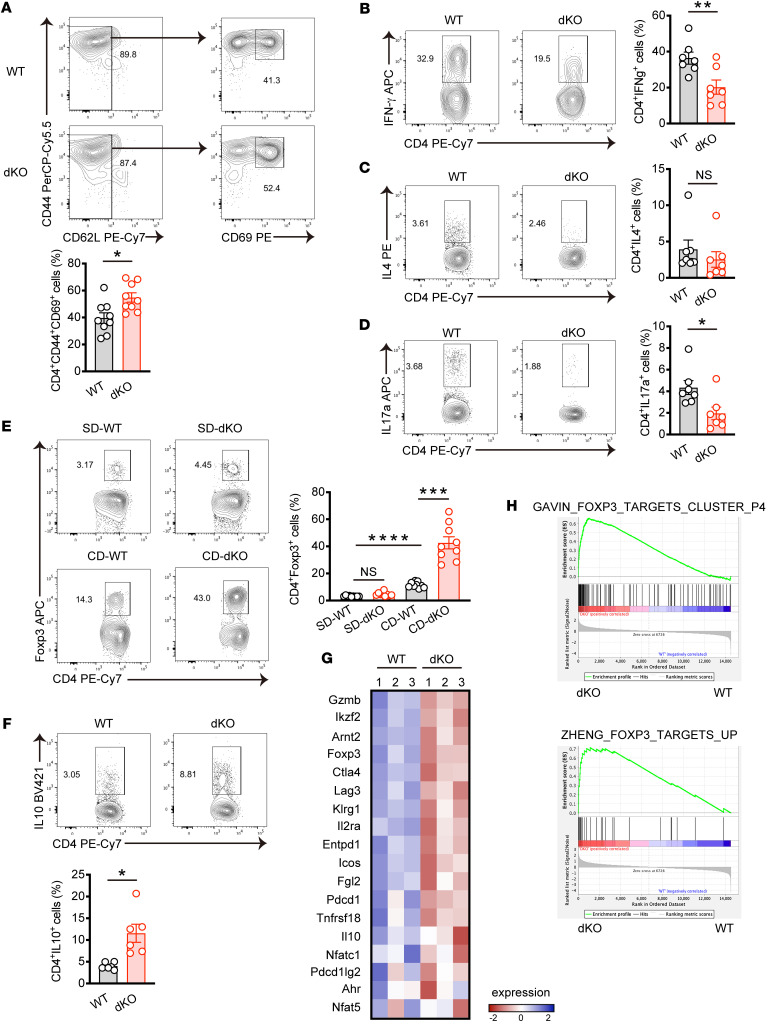
Loss of *Nr4a1* and *Nr4a2* in T cells promotes hepatic Treg accumulation in MASH. (**A**–**D**) WT and dKO mice were fed CD for 8 weeks. (**A**) Representative flow cytometry plots (top) and percentages (bottom) of CD44^+^CD62L^–^/^lo^CD69^+^ cells gated on hepatic CD45^+^CD4^+^ cells (*n* = 9 per group). (**B**–**D**) Representative flow cytometry plots (left) and percentages (right) of IFN-γ^+^ (**B**), IL-4^+^ (**C**), and IL-17a^+^ (**D**) cells gated on CD45^+^CD4^+^ cells in the liver (*n* = 7 per group). The cells were stimulated with phorbol 12-myristate 13-acetate (PMA) and ionomycin for 5 hours in the presence of Golgi plug before staining indicated cytokines. (**E**) WT and dKO mice were fed SD or CD for 8 weeks (*n* = 9 per group). Representative flow cytometry plots (left) and percentages (right) of Foxp3^+^ cells gated on CD45^+^CD4^+^ cells in the liver. (**F**) WT and dKO mice were fed CD for 8 weeks (*n* = 6 per group). Representative flow cytometry plots (top) and percentages (bottom) of IL-10^+^ cells gated on CD45^+^CD4^+^ cells in the liver. The cells were stimulated and stained as in **B**–**D**. (**G** and **H**) RNA-Seq was performed using hepatic CD4^+^ T cells sorted from WT and dKO mice fed CD for 8 weeks (*n* = 3 per group). (**G**) Heatmap of Treg-related genes expressed between WT and dKO hepatic CD4^+^ T cells. (**H**) GSEA of previously published Tregs features enrichment in WT and dKO hepatic CD4^+^ T cells. *P* values were calculated using unpaired 2-tailed Student’s *t* test or Mann-Whitney *U* test (**A**–**D** and **F**) or 1-way ANOVA (**E**). *P < 0.05; **P < 0.01; ***P < 0.001; ****P < 0.0001.

Next we investigated how the T cell Nr4a family impacts MASH development. Despite the massive expansion of dKO CD8^+^ T cells in the context of MASH resistance observed in dKO mice, this seems to be somewhat contradictory, as previous studies demonstrated the involvement of hepatic CD8^+^ T cells in MASH pathology (12, 13). To clarify this, we determined whether CD8^+^ T cell Nr4a affected MASH progression using CD8^+^ T cell–specific *Nr4a1*- and *Nr4a2*-deficient mice (*Cd8Cre* dKO mice). However, histological analysis revealed that *Cd8Cre* dKO mice and WT mice showed similar levels of CD-induced liver fibrosis (Supplemental Figure 4, A and B). Likewise, expression levels of fibrosis-related genes were comparable between the 2 groups (Supplemental Figure 4C). These data suggest that dKO CD8^+^ T cells may not contribute to the attenuated liver fibrosis phenotype observed in dKO mice. Consequently, we redirected our focus toward understanding the functions of the Nr4a family in hepatic CD4^+^ T cells. We first examined whether metabolic stress caused by CD affects CD4^+^ T cell differentiation. T helper cell master transcription factors, except *Tbx21*, were upregulated in hepatic CD4^+^ T cells from mice with MASH. Of note was the significant upregulation of *Foxp3*, which regulates the differentiation and function of Tregs, indicating the involvement of this population in MASH progression (Supplemental Figure 4D). Although no significant differences were observed with respect to total CD4^+^ T cell frequency and number between WT and dKO cells (Figure 3B and Supplemental Figure 3D), tissue-resident memory (CD44^hi^CD62L^lo/–^CD69^+^) CD4^+^ T cells were increased in dKO cells in MASH (Figure 4A). While T helper 2 (Th2) (CD4^+^IL4^+^) cell frequencies were similar in dKO CD4^+^ T cells compared with WT CD4^+^ T cells, Th1 (CD4^+^IFN-γ^+^) and Th17 (CD4^+^IL17A^+^) cells were reduced (Figure 4, B–D). The remarkable change was that dKO hepatic CD4^+^ T cells showed increase of both frequency and cell number of Tregs (CD4^+^Foxp3^+^) in MASH (Figure 4E and Supplemental Figure 4E). In accordance with this, elevated IL-10–producing CD4^+^ T cells were found in MASH dKO mice (Figure 4F). Importantly, Treg populations in the spleen and cervical lymph node showed no significant differences between WT and dKO mice fed CD (Supplemental Figure 4F), suggesting that Treg expansion observed in dKO mice was specific to the liver tissue. To corroborate these findings, we sorted CD4^+^ T cells from liver with MASH and performed bulk RNA-Seq analysis. Based on a cut-off of a fold change greater than 1.5 and less than 0.5 with *P* < 0.05, we identified 560 significantly differentially expressed genes, of which 475 genes were upregulated and 85 genes were downregulated in dKO CD4^+^ T cells compared with WT CD4^+^ T cells (Supplemental Figure 4G). Transcriptional profiling of CD4^+^ T cells revealed a pronounced skew toward Tregs in dKO CD4^+^ T cells showing higher expression of *Foxp3*, *Ikzf2*, and *Ctla4* compared with WT CD4^+^ T cells (Figure 4G). We further performed gene set enrichment analysis (GSEA) and found that IL-2 STAT5 signaling, which is essential for Treg differentiation, was upregulated in dKO CD4^+^ T cells (Supplemental Figure 4H). In addition, genes upregulated in dKO CD4^+^ T cells were highly enriched for the Treg signature (29, 30) (Figure 4H). We next investigated whether these findings were recapitulated in the loss of Nr4a1 and Nr4a2 in T cells after induction of MASH. To this end, *Nr4a1^flfl/fl^Nr4a2^fl/fl^Cd4Cre-ER^T2^* mice, which express tamoxifen-inducible Cre recombinase in T cells, were subjected to a 6-week CD, followed by tamoxifen injection for 5 consecutive days and subsequent analysis after 6 weeks (Supplemental Figure 4I). Histological analysis demonstrated an attenuation of liver fibrosis in *Nr4a1^fl/fl^Nr4a2^fl/fl^Cd4Cre-ER^T2^* mice (idKO mice) (Supplemental Figure 4J). In addition, flow cytometric analysis revealed a reduction of infiltrated macrophages, and IL-17a–producing Th17 cells were found in the liver of idKO, mice whereas dKO Tregs were increased (Supplemental Figure 4, K–N). Altogether, these data suggest that loss of the Nr4a family in T cells promotes hepatic Treg expansion in liver inflammation caused by metabolic stress, and Tregs might be implicated in the process of MASH development.

### dKO Tregs are clonally expanded during MASH progression.

**Figure 5:**
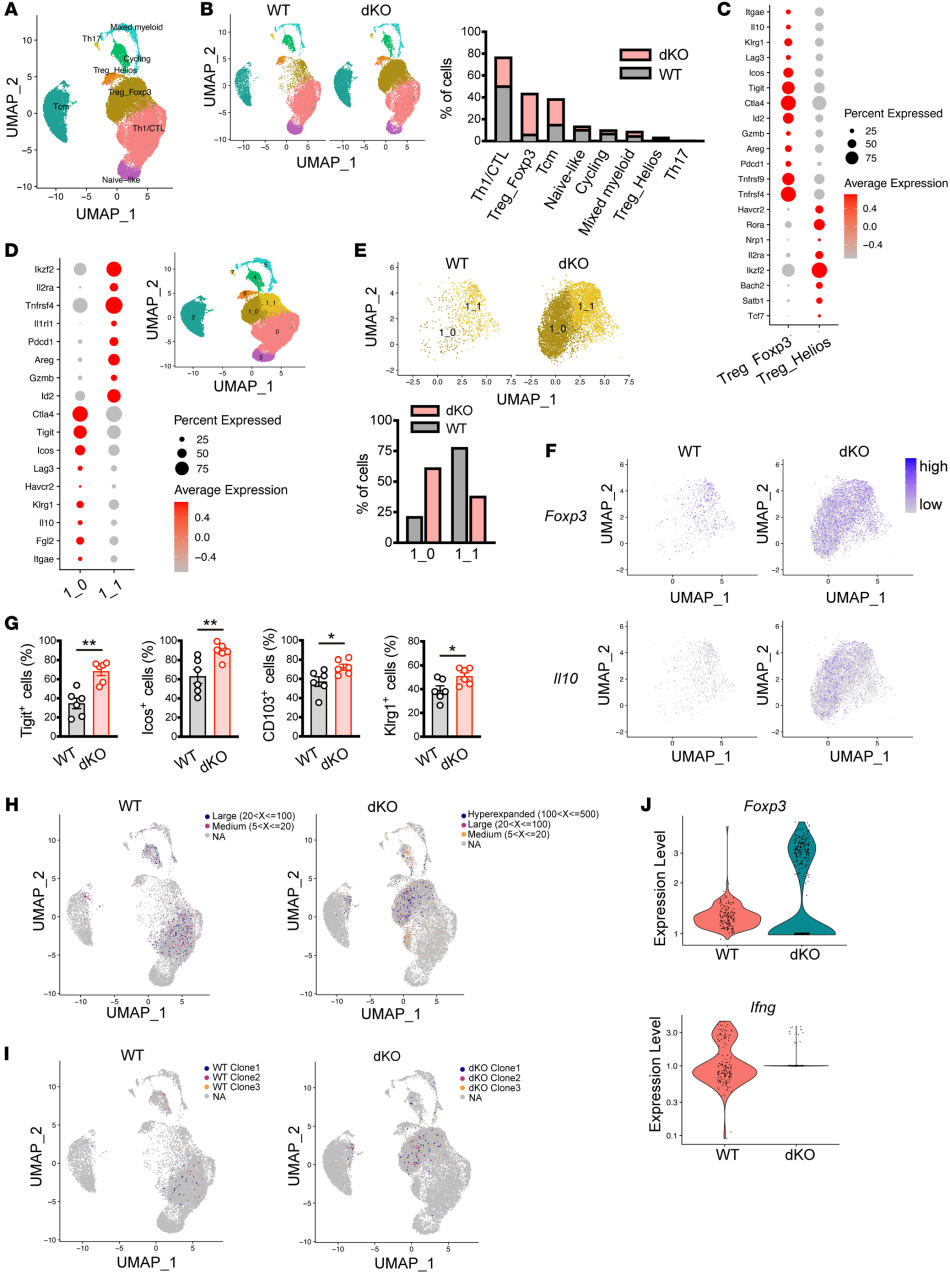
scRNA-Seq coupled with scTCR-Seq analysis identifies clonally expanded hepatic dKO Tregs in MASH. (**A**) UMAP presentation of identified cell populations of hepatic CD4^+^ T cells of male WT and dKO mice fed CD for 8 weeks (*n* = 2 per group). (**B**) UMAP presentation (left) and frequencies (right) of cells in each cluster from WT and dKO hepatic CD4^+^ T cells. (**C**) Dot plot showing expression of indicated genes between Treg_Foxp3 and Treg_Helios clusters. (**D**) Dot plot showing expression of indicated genes between subclusters (left). UMAP presentation of 2 subclusters within Treg_Foxp3 cluster (right). (**E**) UMAP presentation of 2 subclusters within Treg_Foxp3 cluster (top) and frequencies of cells in each subcluster from WT and dKO hepatic CD4^+^ T cells (bottom). (**F**) UMAP presentation showing expression levels of *Foxp3* (top) and *Il10* (bottom) in Treg_Foxp3 cluster of WT and dKO hepatic CD4^+^ T cells. (**G**) Percentages of Tigit^+^, Icos^+^, CD103^+^, and Klrg1^+^ cells gated on hepatic CD45^+^CD4^+^Foxp3^+^ cells from WT and dKO mice fed CD for 8 weeks (*n* = 6 per group). (**H** and **I**) Spatial distribution of expanded clonotypes (**H**) and top 3 clonotypes expanded (**I**) in WT and dKO hepatic CD4^+^ T cells from mice fed CD for 8 weeks. (**J**) Violin plot of gene expression of *Foxp3* (top) and *Ifng* (bottom) in top 3 clonotypes expanded from WT and dKO hepatic CD4^+^ T cells in MASH mice. *P* values were calculated using unpaired 2-tailed Student’s *t* test or Mann-Whitney *U* test (**G**). *P < 0.05; **P < 0.01.

To further dissect the cellular composition of hepatic CD4^+^ T cells in MASH, we performed combined scRNA and single-cell TCR (scTCR) sequencing of hepatic CD4^+^ T cells from WT and dKO mice fed CD. All data sets were aligned using 10X Genomics Cell Ranger pipelines, and clustering was performed using the Seurat package (31). After filtering the scRNA-Seq data, a total of 27,979 cells were visualized using a uniform manifold approximation and projection (UMAP) analysis and clustered into 8 distinct populations (Figure 5A). Cell identities for each cluster were annotated based on differential expression of canonical marker genes (Supplemental Figure 5A): The Th1/CTL cluster represented the largest population and was heterogenous with *Ifn*g and *Nkg7* expression. Based on the expression of *Foxp3* and *Ikzf2*, we identified 2 Treg clusters, Treg_Foxp3 and Treg_Helios. Tcm cluster, which had high expression of *Cd44* and *Cd69*, represented central memory T cell characteristics, while naive-like cluster showed the highest expression of *Tcf7*, *S1p1r*, and *Ccr7*. The cycling cluster had high expression of *Mki67*, which encodes a nuclear protein expressed in proliferating cells. We identified Mixed_myeloid cluster showing the highest expression of representative myeloid cell marker genes *Lyz2* and *Apoe* as previously reported in CD8^+^ T cell transcriptome analysis (32). Finally, a Th17 cell population that expresses *Ccr6*, *Rorc*, and *Il23r* was detected. In comparison between WT and dKO cells, 10,541 cells from WT and 17,438 cells from dKO mice were obtained. Spatial distribution of hepatic CD4^+^ T cells was not affected between WT and dKO CD4^+^ T cells, whereas dKO cells exhibited an increase in the frequencies of cells in Treg_Foxp3 and Tcm clusters and reduction of the Th1/CTL cluster (Figure 5B). These results further confirmed prominent expansion of dKO Tregs in the liver of MASH mice. Thus, we focused our attention on investigating the Treg population. We noticed that T cell immunoreceptor with Ig and ITIM domains (*Tigit*) and *Ikzf2* expression were in sharp contrast between Treg_Foxp3 and Treg_Helios (Figure 5C and Supplemental Figure 5B). Moreover, monocle pseudotime analysis demonstrated developmental trajectory from the Treg_Helios to the Treg_Foxp3 cluster (Supplemental Figure 5C), suggesting that the Treg_Foxp3 cluster contains more mature and activated Tregs. Interestingly, when we adaptively transferred naive CD4^+^ T cells into *Rag2^–/–^* mice, a minor but discernible population of Tregs within transferred CD4^+^ T cells in the liver of both SD- and CD-fed recipients was observed (Supplemental Figure 5, D and E). Notably, recipients fed with CD showed higher frequency of hepatic Tregs compared with those fed with SD (Supplemental Figure 5E). These data indicate that hepatic Tregs mainly originate from thymus as Foxp3^+^CD4^+^ T cells, while to a lesser extent, they also arise from the conversion of naive CD4^+^ T cells in the periphery (18). To gain detailed characterization of Treg populations, the Treg_Foxp3 cluster was divided into 2 subclusters (1_0 and 1_1 cluster) by principal component analysis–based (PCA-based) clustering. We identified clustered cells by their expression of canonical Treg marker genes. The 1_0 cluster had high expression of *Tigit*, *Ctla4*, *Il10*, and *Fgl2*, all of which were shown to be expressed on immunosuppressive Tregs (33). We found high expression of *Areg*, *Il1rl1*, and *Ikzf2* in the 1_1 cluster and these genes are feature of Tregs that function in tissue repair upon muscle injury (34). Interestingly, gene expression profiles between the 1_0 and 1_1 cluster were mutually exclusive, suggesting that hepatic Foxp3-expressing Tregs were heterogenous (Figure 5D). Less than 25% of the Treg_Foxp3 population was composed of the 1_0 cluster in WT cells, while dKO cells had twice as many as WT cells (Figure 5E), suggesting that dKO Tregs acquired greater immunosuppressive competence. Within the dKO Treg_Foxp3 cluster, marked increases in *Foxp3-* and *Il10*-expressing cells were observed (Figure 5F). To further investigate this, we next reconstructed the trajectory of 2 Treg clusters, Treg_Foxp3 and Treg_Helios. Since the Treg_Helios cluster contained less mature Tregs, we designated this cluster as the root of pseudotime and analyzed transcriptional continuum of genes along with pseudotime. *Foxp3* expression was relatively low at the earlier stage, induced at the intermediate stage, then gradually dropped in WT cells. However, expression of *Foxp3* in dKO cells was higher than in WT cells even at the start point and maintained over pseudotime. *Klrg1* expression was inducible in both WT and dKO cells, while being sustained in dKO cells. Expression levels of *Tigit* consistently stayed higher in dKO cells, whereas WT cells expressed *Il10* at substantially lower levels (Supplemental Figure 5F). These results from scRNA-Seq data were validated using flow cytometry analysis, revealing upregulation of cell-surface molecules highly expressed in the 1_0 cluster. (Figure 5G). Notably, frequencies of Tigit^+^ Tregs were similar between WT and dKO splenocytes from mice fed CD, suggesting local expansion of dKO Tregs in the liver during MASH (Supplemental Figure 5G). We next analyzed the TCR repertoire data obtained from scTCR sequencing data using scRepetoire (35). Although both WT and dKO CD4^+^ T cells exhibited largely low-frequency clonotypes, highly expanded clones were more prevalent in dKO cells (Supplemental Figure 5H). Consistent with this, reduction of clonal diversity as measured by 5 distinct metrics was observed in dKO T cells (Supplemental Figure 5I). These data suggest that loss of Nr4a1/2 in T cell promotes clonal expansion of hepatic CD4^+^ T cells during MASH development. We then analyzed clonotype sharing between WT and dKO cells. As a result, clonal expansion scatter plots showed individual expansions in most clonotypes (Supplemental Figure 5J). These finding demonstrate a distinct clonal expansion pattern between WT and dKO CD4^+^ T cells in MASH. We next integrated scTCR-Seq data with scRNA-Seq data to trace the expanded clones. Expanded clones were identified in distinct clusters between WT and dKO cells; WT clones were distributed to the Th1/CTL cluster. On the other hand, dKO cells contained highly expanded clones, primarily within the Treg_Foxp3 cluster. It should be noted that these highly expanded clones in dKO cells were mainly restricted to the 1_0 cluster (Figure 5H). Similar results were obtained from the top 3 clones in each group (Figure 5, I and J). Overall, these data suggest that loss of Nr4a1/2 in T cells promotes expansion of hepatic Tregs, particularly, that possess high potency for antiinflammation.

### Nr4a1 and Nr4a2 inhibit Treg proliferation.

**Figure 6:**
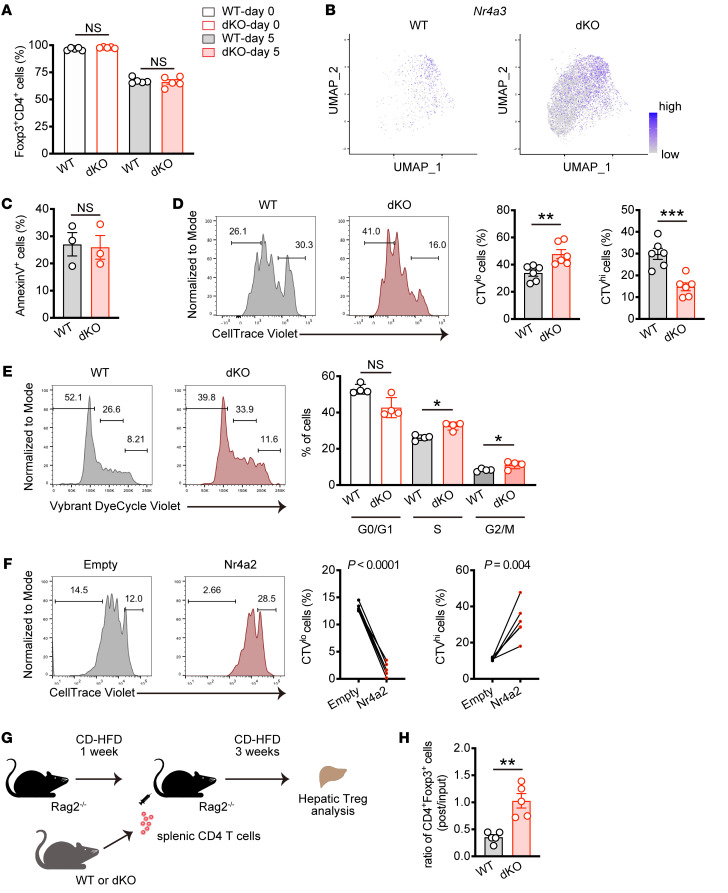
Loss of *Nr4a1* and *Nr4a2* in T cells promotes Treg proliferation. (**A**) Percentages of Foxp3-expressing cells in sorted splenic CD4^+^CD25^+^ Tregs (day 0) and cultured cells for 5 days (day 5) (*n* = 5 per group). (**B**) UMAP presentation showing *Nr4a3* expression within Treg_Foxp3 cluster in WT and dKO hepatic CD4^+^ T cells from mice fed CD for 8 weeks. (**C**) Percentages of Annexin V^+^ WT and dKO splenic Tregs cultured after 3 days (*n* = 3 per group). (**D**) Representative CTV intensity histograms (left) and percentages of CTV^lo^ (middle) and CTV^hi^ (right) cells in WT and dKO splenic Tregs. Sorted Tregs were labeled and cultured for 3 days (*n* = 6 per group). (**E**) Representative flow cytometry analysis of cell cycle distribution (left) and quantification (right) in cultured WT and dKO splenic Tregs. After 3 days, cultured cells were labeled with Vybrant DyeCycle violet (*n* = 4 per group) and analyzed. (**F**) Representative CTV intensity histograms (left) and percentages of CTV^lo^ (middle) and CTV^hi^ (right) cells in splenic Tregs transduced with an empty vector or a vector encoding Nr4a2. Sorted Tregs were retrovirally transduced, rested, labeled with CTV, and then restimulated with α-CD3 and α-CD28 antibodies for 3 days. The cells were gated on GFP^+^CD4^+^ cells (*n* = 6 per group). (**G**) Schematic representation of adaptive transfer of WT and dKO splenic CD4^+^ T cells into MASH-induced *Rag2^–/–^* mice. (**H**) The ratio of hepatic CD4^+^Foxp3^+^ cell frequencies (post) from recipient mice fed CD to splenic CD4^+^Foxp3^+^ cell frequencies (input) from WT and dKO mice (*n* = 5 per group). *P* values were calculated using unpaired 2-tailed Student’s *t* test or Mann-Whitney *U* test (**A**, **C**–**E**, and **H**) or paired 2-tailed Student’s *t* test (**F**). *P < 0.05; **P < 0.01; ***P < 0.001.

To elucidate the mechanisms by which the Nr4a family controls Treg expansion, we investigated Treg homeostasis in vitro. First, we sorted CD4^+^CD25^hi^ Tregs from the spleen and cultured them for 5 days in the presence of IL-2, then analyzed Foxp3 stability. After cell sorting, more than 95% of cells expressed Foxp3, and 5 days of culture resulted in reduction of Foxp3 expression. In this setting, there was no significant difference between WT and dKO Tregs (Figure 6A and Supplemental Figure 6A). Similar results were obtained even after long-term culture (Supplemental Figure 6B). Notably, scRNA-Seq analysis revealed strong upregulation of *Nr4a3* in dKO Tregs (Figure 6B). Together, these data indicate that Nr4a3 alone is sufficient to induce and maintain Foxp3 expression. We next asked whether the Nr4a family regulates Treg survival or proliferation. While the viability of Tregs was similar between 2 groups, as shown by annexin V staining, CellTrace Violet (CTV) assay demonstrated promoted cell division in dKO Tregs (Figure 6, C and D), indicating that Nr4a1/2 limits Treg proliferation but not cell death. This was supported by acute deletion of *Nr4a1* and *Nr4a2* after exposure of *Nr4a1*- and *Nr4a2*-floxed, Cre-ER^T2^–expressing Tregs to 4-hydroxytamoxifen, which resulted in increased cell proliferation (Supplemental Figure 6C). Furthermore, we assessed the cell cycle distribution of Tregs with a DNA-labeling dye and found that more dKO Tregs were detected in the S and G2/M phase compared with WT Tregs (Figure 6E). Consistent with this, we also found increased cycling cells in hepatic Treg population in MASH dKO mice (Supplemental Figure 6D). In contrast, Nr4a2 overexpression in Tregs led to a severe proliferation defect (Figure 6F). Finally, we investigated whether these observations were due to CD4^+^ T cell–intrinsic function of the Nr4a family in Treg proliferation. To address this, we used Rag2^–/–^ mice lacking lymphocytes, fed them CD for 1 week, then adaptively transferred CD4^+^ T cells isolated from WT or dKO mice, followed by another 3 weeks of CD feeding (Figure 6G). As a result, frequency of Foxp3^+^ Tregs decreased in WT cells after transfer, while they were retained in dKO cells (Figure 6H). Taken together, these data suggest that Nr4a1/2 negatively regulates Treg proliferation in response to TCR stimulation, which may control MASH progression.

### The Nr4a/Batf axis functions in Treg proliferation.

**Figure 7:**
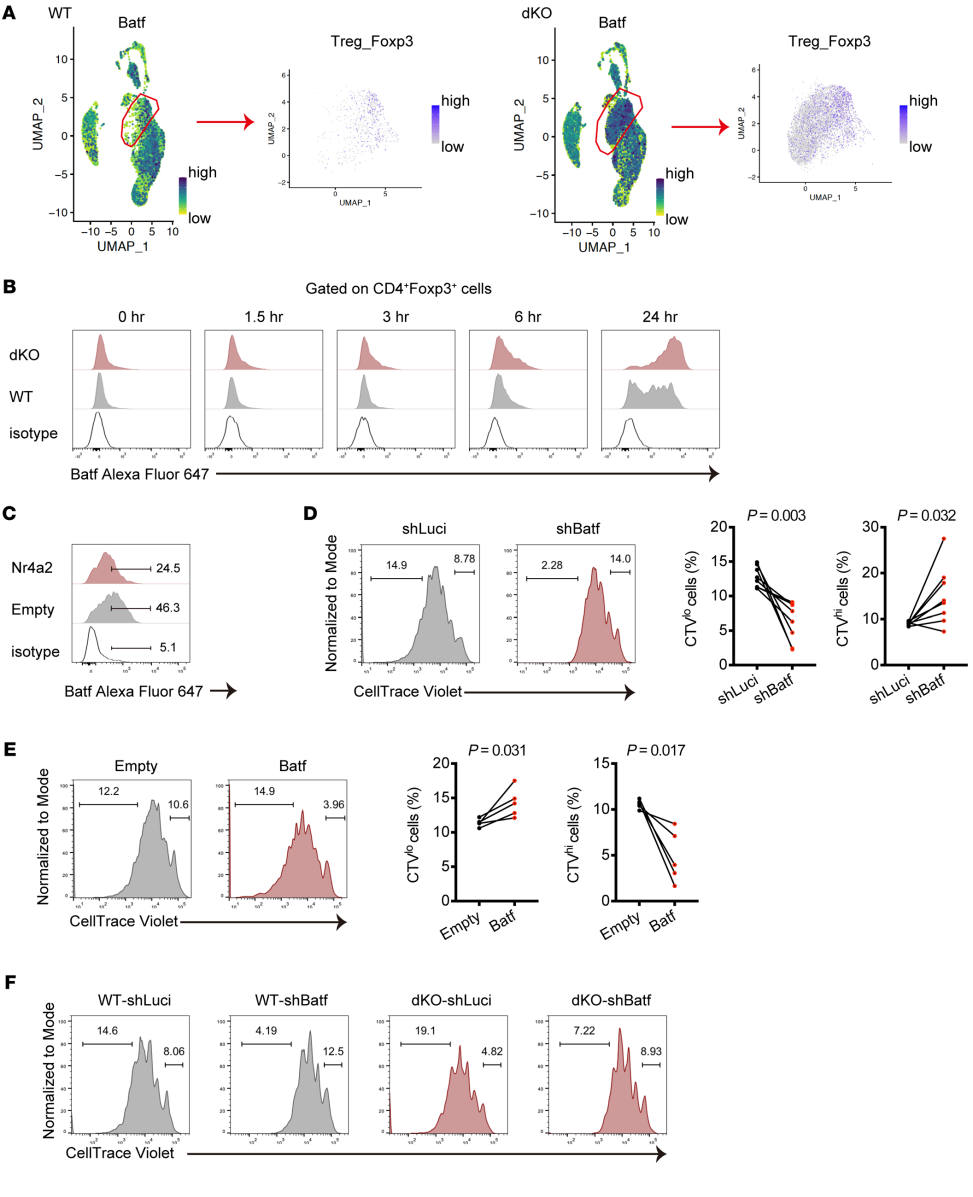
Nr4a/Batf axis regulates Treg proliferation. (**A**) UMAP presentation showing *Batf* expression in assigned all clusters (left) and Treg_Foxp3 cluster (right) from WT and dKO CD4^+^ T cells in the liver of MASH mice. (**B**) Representative histograms normalized to mode for Batf expression in WT and dKO CD4^+^Foxp3^+^ T cells from 2 independent experiments. Sorted splenic CD4^+^ T cells were stimulated with plate-bound α-CD3 antibodies for the indicated times. (**C**) Representative histograms normalized to mode for Batf expression in splenic Tregs transduced with an empty vector or a vector encoding Nr4a2 from 2 independent experiments. Sorted Tregs were retrovirally transduced, rested, and then restimulated with α-CD3 and α-CD28 antibodies for 24 hours. The cells were gated on GFP^+^CD4^+^ cells. (**D**) Representative CTV intensity histograms normalized to mode in splenic Tregs transduced with shRNA targeting *Luciferase* or *Batf* (left) and percentages of CTV^lo^ (middle) and CTV^hi^ (right) cells. After resting culture, the cells were labeled and restimulated with α-CD3 and α-CD28 antibodies for 3 days. The cells were gated on GFP^+^CD4^+^ cells (*n* = 8 per group). (**E**) Representative CTV intensity histograms normalized to mode in splenic Tregs transduced with an empty vector or a vector encoding Batf (left) and percentages of CTV^lo^ (middle) and CTV^hi^ (right) cells. After resting culture, the cells were labeled and restimulated with α-CD3 and α-CD28 antibodies for 3 days. The cells were gated on GFP^+^CD4^+^ cells (*n* = 5 per group). (**F**) Representative CTV intensity histograms normalized to mode in WT and dKO splenic Tregs transduced with shRNA targeting *Luciferase* or *Batf* from 2 independent experiments. The cells were gated on GFP^+^CD4^+^ cells. *P* values were calculated using paired 2-tailed Student’s *t* test (**D** and **E**).

To gain insight into the clonal expansion of dKO Tregs in MASH, we sought the downstream target genes of the Nr4a family in Tregs in our scRNA-Seq data. We noted that a transcription factor, basic leucine zipper ATF-like transcription factor (Batf), which has been shown to play a crucial role for Treg development in the tumor microenvironment (36), was expressed on the Tregs_Foxp3 cluster that was remarkably expanded in dKO cells (Supplemental Figure 7A). We found that upregulated *Batf* expression was observed in the Tregs_Foxp3 cluster in dKO cells (Figure 7A). Trajectory analysis of the Treg_Foxp3 and Treg_Helios clusters revealed that *Batf* transcription in dKO cells was strongly induced and maintained at high levels even in the later stage of pseudotime (Supplemental Figure 7B). In contrast, enforced Nr4a2 expression in CD4^+^ T cells suppressed *Batf* gene expression (Supplemental Figure 7C). Using flow cytometry analysis, we validated whether the Nr4a family regulates Batf expression in Tregs at protein levels upon activation. We stimulated CD4^+^ T cells with plate-coated α-CD3 antibody and observed that Batf protein was induced at 6 hours after stimulation in WT Foxp3^+^ Tregs, while dKO Tregs exhibited marked earlier and higher induction of Batf (Figure 7B). Similar results were also obtained when dKO cells were costimulated with α-CD3 and α-CD28 antibodies (Supplemental Figure 7D). Consistently, transduction of Nr4a2 into Tregs resulted in suppression of Batf expression upon stimulation (Figure 7C). We further examined whether the Nr4a family regulates Treg expansion via targeting Batf. We constructed vectors expressing shRNA against *Batf* and found that knockdown of Batf led to a proliferation defect in Tregs (Figure 7D and Supplemental Figure 7E). Conversely, overexpression of Batf in Tregs significantly decreased undivided cells (Figure 7E). Finally, we observed that silencing of Batf expression substantially reduced dividing cells in dKO Tregs (Figure 7F). Combined together, these findings suggest that Nr4a1/2 negatively regulates Treg proliferation through suppressing Batf expression upon cell activation.

### Loss of Nr4a1 and Nr4a2 in T cells leads to enhanced suppressive function in Tregs.

**Figure 8:**
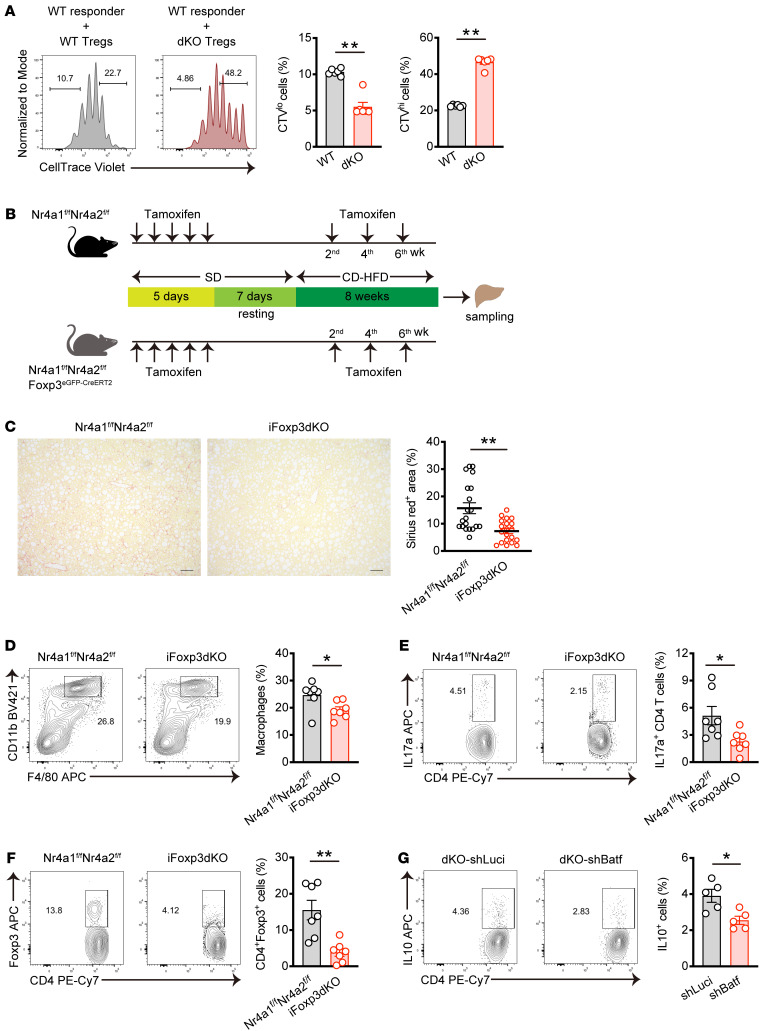
The loss of Nr4a1 and Nr4a2 in T cell promotes Treg function. (**A**) Representative CTV intensity histograms normalized to mode in WT responder cells (left) and percentages of CTV^lo^ (middle) and CTV^hi^ (right) cells. Hepatic CD4^+^CD25^+^Tigit^+^ Tregs were sorted from WT and dKO mice fed CD for 8 weeks. CTV-labeled naive CD4^+^ T cells from the spleen of Ly5.1 mice were activated with Dynabeads Mouse T cell activator CD3/CD28 in the presence of sorted Tregs at a ratio of 5:1 (responder cells:Tregs). The cells were gated on CD45.1^+^CD4^+^ cells (*n* = 6 per group) (**B**) Schematic representation of the experimental procedure of MASH induction mouse model with Treg-specific inducible deletion of Nr4a1 and Nr4a2. (**C**) Representative Sirius red staining of liver sections from female mice. Original magnification, ×10. Scale bars: 100 μm. Quantification of Sirius red staining area (%) per field (right) in MASH-induced WT and iFoxp3dKO mice (*n* = 4 female per group). (**D**–**F**) Representative flow cytometry plots (left) and percentages (right) of macrophages (CD45^+^CD11b^hi^F4/80^int^) (**D**), Th17 cells (CD45^+^CD4^+^IL17a^+^) (**E**), and Tregs (CD45^+^CD4^+^Foxp3^+^) (**F**) in the liver of MASH-induced WT and iFoxp3dKO mice (*n* = 7 per group). (**G**) Representative flow cytometry plots (left) and percentages (right) of IL-10^+^ in dKO splenic Tregs transduced with a shRNA targeting *Luciferase* or *Batf*. After resting culture, the cells were stimulated with PMA and ionomycin for 5 hours in the presence of Golgi plug before staining IL-10. The cells were gated on GFP^+^CD4^+^ cells (*n* = 5 per group). *P* values were calculated using unpaired 2-tailed Student’s *t* test or Mann-Whitney *U* test (**A** and **C**–**G**). *P < 0.05; **P < 0.01.

Our scRNA-Seq data revealed that the frequency of *Ki-67* expressing cells decreased, whereas immunosuppressive subcluster, characterized by high expression of *Il10* and *Ctla4*, increased in dKO cells (Figure 5, B and E). Expanded dKO Tregs in the MASH liver exhibited lower percentages of Ki-67^+^ cells compared with WT Tregs (Supplemental Figure 8A). Therefore, we next investigated whether *Nr4a1* and *Nr4a2* deficiency in T cell impacts the effector functions of Tregs. We isolated hepatic Tregs from MASH mice by sorting CD4^+^CD25^+^Tigit^+^ and performed coculture suppression assay. As shown in Figure 8A, dKO hepatic Tregs showed higher ability to suppress the proliferation of responder naive CD4^+^ T cells. To further examine the effects of Tregs, we crossed *Foxp3^eGFlP–CreERT2^* mice with *Nr4a1^fl/fl^Nr4a2^fl/fl^* mice, allowing for the inducible deletion of *Nr4a1* and *Nr4a2* in Tregs. Prior to MASH induction, mice received intraperitoneal administration of tamoxifen for 5 consecutive days. Subsequently, mice were fed CD for 8 weeks, with tamoxifen given once every 2 weeks during this period (Figure 8B). In the results, *Foxp3^eGFlP–CreERT2^* *Nr4a1^fl/fl^Nr4a2^fl/fl^* (iFoxp3dKO) mice showed attenuated liver fibrosis compared with *Nr4a1^fl/fl^Nr4a2^fl/fl^* mice (Figure 8C). Consistently, we found a reduction in macrophages and IL-17a–producing cells, though not in IFN-γ–producing cells in iFoxp3dKO mice (Figure 8, D and E, Supplemental Figure 8B). Unexpectedly, inducible deletion of *Nr4a1* and *Nr4a2* in Tregs resulted in a reduction of hepatic Tregs, despite the fact that iFoxp3dKO mice exhibited increased resistance to MASH (Figure 8F). These data suggest that dKO led to enhanced immunosuppressive functions in hepatic Tregs during MASH development. To further investigate these findings, we silenced Batf expression using shRNA in dKO Tregs. The results revealed that Batf-downregulated dKO cells produced less IL-10 compared with control cells, indicating that the Nr4a/Batf axis plays a role in regulating suppressive function in Tregs (Figure 8G).

## Discussion

MASH, which is a common clinical disease, is now a major cause of liver dysfunction and there is an urgent need to establish effective therapeutic strategies underpinned by complete understanding of its pathogenesis. In the early stage of MASLD, hepatocytes stressed by steatosis release proinflammatory mediators and damage-associated molecule patterns, which results in the secretion of proinflammatory cytokines and chemokines from hepatic macrophages (11). Given that excessive liver inflammation is widely recognized to be correlated with MASH progression and fibrosis development (2), we investigated how inflammatory responses during MASH are regulated by liver immune cells, particularly focusing on hepatic CD4^+^ T cells.

In recent years, unique populations of Tregs in nonlymphoid tissues such as visceral adipose tissue, skeletal muscle, and skin have been identified as important regulators for tissue homeostasis (18). However, the precise cellular mechanisms by which Tregs regulate inflammatory response in the liver is uncertain. The liver is a heterogeneous organ composed of hepatocytes in addition to diverse nonparenchymal cells, including endothelial cells, macrophages, T cells, B cells, and hepatic stellate cells. Recent advanced transcriptome analysis delineated the landscape of intracellular crosstalk in healthy and MASH liver (9, 37). We found that dKO mice contained less hepatic Ly-6C^+^ macrophages, which are crucial for inflammatory response and fibrosis (24, 38). Furthermore, hepatic CD11b^+^ cells in dKO mice displayed elevated expression of *Arg1* and *Nos2*, both known to be associated with tolerogenic characteristics (28). Meanwhile, the marker gene of MASH-associated M1 macrophages, *Trem2*, was highly expressed in CD11b^+^ cells from WT mice (7, 25). These data indicate that Tregs may directly or indirectly regulate macrophage polarization, shifting them from proinflammatory to antiinflammatory phenotypes. This notion is supported by a previous study, suggesting that Tregs in muscle tissue reduce CD11b^+^Ly-6c^hi^ myeloid cells that infiltrate damaged tissue (34). Alternatively, IL-10–producing Tregs may enhance macrophage function, particularly in terms of apoptotic cell engraftment during resolution of inflammation (39).

We also found a decrease of the hepatic Th17 subset in dKO mice. In both humans and mice with MASH, increased frequencies of hepatic Th17 cells were observed and the Th17/Treg ratio was higher in patients with MASH than MASLD (40). Inflammatory hepatic CXCR3^+^ Th17 cells have been identified as the driver for MASH development (41), while lacking *Il17* in mice attenuated hepatic inflammation (41–43). Thus, it is also plausible that expanded Tregs in dKO mice may selectively suppress proinflammatory Th17 cell response.

In this study, we conducted scRNA-Seq analysis of hepatic CD4^+^ T cells in the MASH mouse. This approach provides insights into the spectrum of heterogeneity among CD4^+^ T cells during MASH progression. Two distinct clusters, Treg_Foxp3 and Treg_Helios, were identified as Treg subsets based on their high expression of *Foxp3*. The Treg_Helios cluster characterized with high expression of *Bach2* and *Satb1* and low expression of *Id2* appeared to represent the resting Tregs subset (44). Considering the higher expression of *Ikzf2* and *Nrp1* in Treg clusters, hepatic Tregs were likely derived from thymus rather than being converted from Foxp3^–^CD4^+^ T cells in the periphery (18). However, we observed minor Foxp3^+^ T cells within transferred naive CD4^+^ T cells in the liver of *RAG2^–/–^* mice, suggesting a potential origin from Foxp3^–^CD4^+^ T cells through peripheral conversion. While the origin of hepatic Tregs remains elusive, our data suggest that there are distinct mechanisms governing their development to regulate liver homeostasis. In our trajectory analysis, hepatic Treg clusters showed developmental connectivity illustrating the directional flow from the Treg_Helios to Treg_Foxp3 cluster. Indeed, the Treg_Foxp3 cluster had high expression of genes related to suppressive function of Tregs, such as *Tigit*, *Ctla4*, and *Il10*, indicative of mature Tregs phenotype (45). Further subclustering of the Treg_Foxp3 cluster revealed 2 distinct subpopulations. One of these subclusters represented an immunosuppressive population that had expression of *Il10*, *Tigit*, and *Ctla4*, whereas the other was marked by the expression of *Areg* and *Il1rl1*, which are characteristics of tissue repair. TIGIT^+^ Tregs represented in the immunosuppressive population were previously characterized as Tregs endowed with specific suppressor activities for Th1 and Th17 cell response (33).Notably, frequency of this subpopulation was more prominent in dKO cells than WT cells, further supporting the notion that dKO Tregs acquire enhanced effector functions and exert dampening effect on local inflammation, provably suppressing Th1/Th17 responses within MASH liver.

A recent advance in single-cell transcriptome enables simultaneous analysis of transcriptome and clonotype information by integration of scTCR-Seq with scRNA-Seq. This innovative approach is useful to dissect the clonal expansion dynamics of T cells and track the differentiation trajectory of the clones (32, 46, 47). Our findings indicate that dKO CD4^+^ T cells exhibited higher levels of clonal expansion compared with WT CD4^+^ T cells. Additionally, a substantial portion of this clonal expansion was correlated with the subcluster displaying *Tigit* expression in the Treg_Foxp3 cluster, further supporting the ameliorated liver inflammation in dKO mice. Future studies will be needed to investigate the antiinflammatory effect of these TCR repertoires during MASH progression. Although a small fraction of the dKO clones that highly expanded in the Tregs_Foxp3 cluster was also identified in different clusters such as the Tcm cluster, it remains uncertain whether these expanded Tregs were derived from small populations in another cluster or directly recruited from lymphoid organs in response to local inflammation. On the other hand, WT cells showed a relatively higher diversity of clonotypes compared with dKO cells and expanded clonotypes did not mostly overlap with those in dKO cells. Moreover, expanded clonotypes of WT cells were predominantly associated with the Th1/CTL cluster that had high *Ifng* expression. Thus, expanded CD4^+^ T cells between WT and dKO cells did not share the common TCR repertoire and could be distinguished as distinct subsets. One can speculate that the expanded clonotypes within the Treg_Foxp3 cluster might selectively target the clones distributed to the Th1/CTL cluster, and the balance among those clonotypes could be potentially associated with severity of liver inflammation and MASH.

We observed that dKO Tregs underwent local expansion in inflamed tissue, with no such expansion observed in the spleen and cervical lymph node. This phenomenon was recapitulated in the liver of *Rag2*^–/–^ recipients with MASH adaptively transferred dKO CD4^+^ T cells, indicative of CD4^+^ T cell–intrinsic mechanisms. Surprisingly, similar local expansion in dKO Tregs was not seen in the liver of iFoxp3dKO mice, despite observed reduced liver fibrosis. One plausible speculation is that Nr4a1 and Nr4a2 deficiency in Tregs initially promotes cell proliferation due to heightened responsiveness to TCR stimulation. Subsequently, this enhances the suppressive capability, eventually leading to a self-limiting proliferation by Tregs themselves. Supporting this hypothesis, hepatic dKO Tregs showed lower Ki-67 expression and heightened suppressive activity in vitro. Therefore, it is conceivable that conventional T cells also contribute to adaptation and local accumulation of the Tregs pool in the MASH liver. Our data imply the underlying mechanisms governing the population dynamics of Tregs, involving not only a Treg-intrinsic mechanism, but also through communication among distinct T cell populations in the MASH liver.

Our investigation into the underlying mechanism revealed the involvement of the Nr4a/Batf axis in regulating Treg proliferation and functions. Batf, a transcription factor belonging to the AP-1/ATF superfamily, is shown to be selectively expressed on skin and adipose tissue Tregs that have regenerative characteristics (48). Nr4a2 transduction into Tregs suppressed Batf expression, whereas dKO Tregs exhibited stronger induction upon TCR stimulation, suggesting that the Nr4a family acts as a repressor of Batf, consistent with our previous study from TGF-β–induced Tregs in vitro (49). Although it has been demonstrated that Batf controls CD8^+^ T cell expansion in tumor or chronic virus infection, the biological outcomes are likely context dependent (50–54). Nonetheless, our findings demonstrated that overexpression of Nr4a2 or silencing of Batf reduced Treg proliferation in vitro and indicate that the Nr4a family limits Treg proliferation through downregulating Batf expression. This Nr4a family–mediated circuit of Batf expression seems to be conserved in other cell types, as Nr4a1 and Nr4a3 restrained B cell response in the absence of costimulation through repression of Batf expression (55). Mice lacking *Batf* showed a reduction of tissue-infiltrating Tregs (22, 36, 56, 57), suggesting the requirement of Batf in tissue Treg development. An integrative transcriptome analysis demonstrated that Batf bound to loci of *Ctla4*, *Tigit*, and *Il10*, genes whose expression was elevated in hepatic dKO Tregs (36). Importantly, RNAi-mediated gene silencing of *Batf* in dKO Tregs reduced IL-10 production in vitro. Moreover, our trajectory analysis unveiled sustained high expression levels of these genes, along with *Foxp3* throughout the pseudotime in dKO Tregs. While loss of Nr4a1/2 did not affect Foxp3 expression in cultured Tregs, our data imply that the Nr4a/Batf circuit may also be pivotal for tissue Treg development and maintenance in vivo, particularly within an inflammatory environment.

It should be noted that there is a limitation in this study regarding its applicability to human MASH. Although previous studies demonstrated an increase in hepatic Tregs in humans with MASLD/MASH, similar to observations in mice (58, 59), the efficacy of Tregs in patients with MASLD/MASH has not yet been examined in a clinical setting. In mice, it has been shown that Tregs can positively or negatively control MASH progression, indicating dual antiinflammatory and profibrotic activities of hepatic Tregs during MASLD/MASH progression (15, 59, 60). We found an increase in Foxp3^+^ Tregs, which exert suppression of inflammation among CD4^+^ T helper cell subsets in the liver of MASH mice, consistent with previous reports (16, 17). We also discovered that dKO mice exhibited alleviated MASH pathology, as evidenced by reduced liver injury and fibrosis, accompanied by expanded hepatic Tregs compared with WT mice. Our findings strongly suggest that hepatic Tregs may play a protective role against MASH through enhanced immunosuppressive capability and cell proliferation. Although the therapeutic potential of Nr4a in human cancers has been recently discussed (61), there is currently insufficient evidence demonstrating the involvement of Nr4a receptors in MASH patients. Nonetheless, our findings prompt us to consider the possibility that the adoptive transfer of Tregs expanded ex vivo, targeting the Nr4a/Batf axis, may hold therapeutic promise against MASH.

In conclusion, our study highlights a protective role of hepatic Tregs in MASH pathology and identifies the Nr4a family in T cells as a key driver in the context of MASH. Although T cell–specific deletion of all 3 *Nr4a* genes in mice led to impaired Treg population in thymus and spleen (20), dKO mice did not show such a defect. Loss of *Nr4a1* and *Nr4a2* in Tregs rather promoted cell expansion and functions concomitant with high expression of *Nr4a3*, suggesting that Nr4a3 alone was sufficient for development and maintenance of Tregs. Therefore, targeting the Nr4a family with moderate inhibitors might be a novel and an attractive strategy for mitigating liver inflammation in MASH via enhancing the immune-suppressive effects mediated by Tregs.

## Methods

### Sex as a biological variable.

For the pathological analysis of liver tissues and serum, statistical comparisons were performed between groups with matched sexes, as sex differences have been observed, whereas the findings are expected for both sexes. For cellular-level analyses, sex was not considered, since no significant differences between males and females were observed at this level.

### Animal studies.

*Nr4a1^fl/fl^Nr4a2^fl/fl^* mice, which harbor floxed alleles for *Nr4a1* and *Nr4a2* (20, 62), were crossed with *Cd4-Cre*, *CreER^T2^* (63), *Foxp3^eGFlP–CreERT2^* (64), *Cd4Cre-ER^T2^* (64), or *E8i.Cd8Cre* (*Cd8-Cre)* transgenic mice (65) to generate conditional *Nr4a1*- and *Nr4a2*-deficient mice. C57BL/6-Ly5.1 were obtained from RIKEN BRC. Nr4a3-EGFP mice, which express EGFP under the control of a bacterial artificial chromosomal *Nr4a3* locus, were previously described (66). All mice were bred and maintained in a controlled temperature, humidity, and specific pathogen–free facility under a 12-hour dark/12-hour light cycle with free access to food and water. For MASH diet feeding, the mice were fed l-amino-defined high (60 kcal%) fat diet with 0.1% methionine and no added choline (A06071302, Research Diet) ad libitum for the times indicated in the figure legends. To examine MASH pathology in Figure 1 and Figure 2 and Supplemental Figures 1 and 2, age-matched (6- 8-week-old) male mice were used, unless otherwise stated in the figure legends. For the measurement of serum parameters, sera collected from female mice fed CD for 12 weeks were analyzed for indicated biochemical markers by Oriental Yeast Co. using standard protocols. Total cholesterol levels were determined using L-Type CHO M (Fujifilm), which measures combined LDL and HDL cholesterol. For inducible *Nr4a1* and *Nr4a2* deletion in Tregs, we crossed *Foxp3^eGFlP–CreERT2^* mice with *Nr4a1^fl/fl^Nr4a2^fl/fl^* mice. These mice were intraperitoneally injected with 1 mg of tamoxifen for 5 consecutive days, followed by a 7-day resting period after the last administration. Subsequently, the mice were fed CD for 8 weeks, with tamoxifen administered once every 2 weeks during this period. To examine the effect of inducible depletion of *Nr4a1* and *Nr4a2* in T cells, *Cd4Cre-ER^T2^* mice crossed with *Nr4a1*^fl/fl^*Nr4a2^fl/fl^* mice were subjected to 6 weeks of CD, followed by 1 mg of tamoxifen injection for 5 consecutive days. Mice were further fed CD for an additional 6 weeks and then analyzed. For adaptive transfer experiments, recipient *Rag2*^–/–^ mice (Jackson Laboratories) were fed CD for a week prior to CD4^+^ T cell transfer, received splenic CD4^+^ T cells (2 × 10^6^) from either WT or dKO mice via retro-orbital injection, and then were fed CD for another 3 weeks. As shown in Supplemental Figure 5D, naive CD4^+^ T cells from WT mice were adaptively transferred into *Rag2^–/–^* mice fed either SD or CD.

### Expression constructs, cell culture, and retroviral transduction.

Naive CD4^+^ T cells were sorted and cultured as previously described (67). For Treg culture, splenic CD4^+^CD25^hi^ cells were sorted and stimulated with plate-bound α-CD3 (3 μg/ml, 145-2C11, eBioscience) and α-CD28 (2 μg/ml, 37.51, eBioscience) antibodies for the indicated times in RPMI supplemented with 10% fetal bovine serum, 1 mM sodium pyruvate, 10 mM HEPES, 100 U/ml penicillin, 100 μg/ml streptomycin, 55 μM 2-mercaptoethanol, and 500 U/ml rhIL-2. For retrovirus-mediated gene transfer to T cells, the cDNAs encoding *Batf* were cloned into retroviral vector pMSCV-IRES-GFP (MIGR1, Addgene). To deplete expression of Batf, the oligonucleotides incorporating miR-30 microRNA sequence into the target sequence (provided in Supplemental Table 2) were cloned into retroviral vector pMSCV-LMP (Open Biosystems). Target sequence for firefly luciferase used as control shRNA was described elsewhere (20). These retroviral vectors together with packaging plasmid pCL-ECO were transfected into PLAT-E cells using TransIT-LT1 transfection reagent (MIR2300, Mirus). After overnight incubation, medium was replaced with DMEM medium and culture supernatants containing retroviral particles were collected 48 hours after transfection. Retroviral supernatants were filtrated, supplemented with 8 μg/ml polybrene (TR-1003G, Millipore), and then used to spin infect the cells that previously activated with α-CD3 and α-CD28 antibodies. Spin infection was performed at 2,000 rpm for 60 minutes at 35°C. After 24 hours, cells were washed and rested in the presence of rhIL-2 for another 3 days before restimulation. To induce Cre-ER–mediated recombination and conditionally delete *Nr4a1* and *Nr4a2* in Treg cells, sorted Tregs were stimulated with plate-bound α-CD3 and α-CD28 antibodies in the presence of 500 nM 4-hydroxy-tamoxifen (4-OHT) culture medium for 2 days, then rested for 3 days without 4-OHT before restimulation. For assessment of cell proliferation, cells were labeled with 5 μM CTV dye (C34557, Invitrogen) and stimulated. After 3 days, violet dye dilution was measured by flow cytometry. For cell cycle analysis, cultured Tregs were labeled with 5 μM Vybrant DyeCycle Violet stain (V35003, Invitrogen) and analyzed.

### RNA-Seq.

For bulk RNA-Seq, 3 independent biological replicates were analyzed. Total RNA of hepatic CD4^+^ T cell sorted was extracted and libraries were generated using NEBNext Ultra II FS DNA Library Prep Kit (E7805, NEB). Single end sequencing was performed by NovaSeq 6000 at the ImmunoGeneTeqs. RNA-Seq data were mapped against mouse genome mm10/GRCm39. Differentially expressed genes in dKO cells relative to WT cells were defined as 1.5-fold (upregulated) or 0.5-fold (downregulated) change with *P* value < 0.05, and log_2_ fold changes were shown on the volcano plot. For heatmap generation, transcripts per kilobase million (TPM) values were transformed into row-wise *z* scores. GSEA was performed using the Mouse MSigDB collection (68). For scRNA-Seq, hepatic CD4^+^ T cells were pooled from 2 individual male mice per group and loaded onto a 10X Genomics Chromium instrument to recover 1 × 10^4^ cells. Libraries were constructed using Chromium Next GEM Single Cell 5′ Reagent Kits, version 2 (CG000330, 10X Genomics), according to the manufacturer’s instructions and sequenced using NovaSeq 6000. Raw data from each sample were mapped using the Cell Ranger Multi Pipeline (10X Genomics) to the mm10 reference genome assembly and R package. Seurat was used to analyze data (31). Cells with more than 200 detected genes, less than 5% mitochondrial reads, and genes with transcripts detecting more than 3 cells were used for analysis. The gene counts were log-normalized by Seurat function “NormalizeData” (scale factor 10000) and the top 2000 highly variable genes were identified by Seurat function “FindVariableFeatures”. Expression of variable genes was scaled, and each data set was integrated. Then PCA was performed by Seurat function “RunPCA” to reduce the dimensions of the data. Based on the ElbowPlot, 9 of the principal components were selected and used for unsupervised clustering analysis with resolution parameter of 0.1 by Seurat functions “FindNeighbors” and “FindClusters.” Cell clusters were visualized using UMAP and the identity of each cluster gene was further assigned based on the canonical marker genes. Cell cycle assessment was performed using the CellCycleScoring in Surat, based on the G1, S, and G2/M phase marker expression in each cell in the indicated clusters. The analysis of differentiation trajectories was performed in Monocle3 using the SeuratWrapper package to convert the integrated Seurat object (69). For TCR repertoire analysis, the ScRepertoire package was used to combine the contig data and analyze clonotypes (35). scTCR-Seq data were integrated with the Seurat object of the scRNA-Seq data.

### Statistics.

The data are presented as mean ± SEM. To assess the statistical significance, normal distribution of experimental groups was examined using the Shapiro-Wilk test. Statistical differences between 2 groups were determined by unpaired 2-tailed Student’s *t* test (with or without Welch’s collection), paired *t* test, or Mann-Whitney *U* test. For comparisons of multiple groups, 1-way ANOVA or Kruskal-Wallis test was used. In post hoc analysis, *P* values were corrected by Tukey’s, Dunnett’sT3 multiple-comparison test, or controlling the FDR via the 2-stage linear step-up procedure of Benjamini, Krieger, and Yekutieli. All statistical analysis was performed using GraphPad Prism, version 8.3.1 (GraphPad Software). Details of tests used for each analysis are described in the figure legends. We considered *P* < 0.05 to be significant.

### Study approval.

All animal procedures in this study were approved by the Animal Ethics Committee of Keio University (Japan) and were performed in accordance with the Animal Ethics Committee’s guidelines.

### Data availability.

The RNA-Seq, scRNA-Seq, and scTCR-Seq data sets were deposited in the NCBI’s Gene Expression Omnibus database (GEO GSE241515 and GSE241657). Values for all data points in graphs are reported in the Supporting Data Values file.

## Author contributions

DA conceived and designed the research, conducted the experiments, analyzed the data, and wrote the manuscript. TH analyzed the scRNA Seq data. TS provided the reagents and performed the experiments. SS and MI provided the reagents and acquired the RNA-Seq data. SIS and SMO contributed resources. AY conceived and designed the research and edited the manuscript.

## Supplementary Material

Supplemental data

Unedited blot and gel images

Supporting data values

## Version 1 10/15/2024

In-Press Preview

## Version 2 12/02/2024

Electronic publication
