# Supplementary material for: The Nr4a family regulates intrahepatic Treg proliferation and liver fibrosis in MASLD models
Source: J Clin Invest. 2024 Oct 15;134(23):e175305. doi: 10.1172/JCI175305 (PMC11601941; doi:10.1172/JCI175305)
Supplement: Supplemental data [file jci-134-175305-s124.pdf]

Supplemental Materials

The Nr4a family regulates intrahepatic Treg proliferation and liver fibrosis in MASLD models

Daisuke Aki[1,2*], Taeko Hayakawa[1], Tanakorn Srirat[1], Shigeyuki Shichino[3], Minako Ito[4], Shin-Ichiroh Saitoh[2], Setsuko Mise-Omata[1,5], Akihiko Yoshimura[1,5]

[1]Department of Microbiology and Immunology, Keio University School of Medicine, Tokyo, Japan

[2]Department of Intractable Disorders, Institute of Advanced Medicine, Wakayama Medical University, Wakayama, Japan

[3]Division of Molecular Regulation of Inflammatory and Immune Diseases, Research Institute for Biomedical Sciences, Tokyo University of Science, Chiba, Japan

[4]Division of Allergy and Immunology, Medical Institute of Bioregulation, Kyushu University, Fukuoka, Japan

[5]Research Institute for Biomedical Science, Tokyo University of Science, Chiba, Japan

This file includes the following information;

Supplemental Methods

Supplemental Figure 1 to 8

Supplemental Table 1 to 2

Supplemental Methods

Reagents

For flow cytometry and cell culture, antibodies to CD3e (145-2c11), CD4 (RM4-5), CD8a (53-6.7), CD11b (M1/70), CD25 (PC61.5), CD28 (37.51), CD44 (IM7), CD45 (30-F11), CD62L (MEL-14), CD69 (H1.2F3), CD103 (2E7), B220 (RA3-6B2), F4/80 (BM8), ICOS (C398.4A), KLRG1 (2F1), Ly-6c (HK1.4), NK1.1 (PK136), TIGIT (1G9), IFN-γ (XMG1.2), IL4 (11B11), IL10 (JES5-16E3), IL17a (eBio17B7), Foxp3 (FJK-16s), Ki-67 (SolA15) and Batf (D7C5) were purchased from eBioscience, BioLegend, BD Biosciences and Cell Signaling TECHNOLOGY. For immunoblotting, antibodies to Smooth Muscle Actin (D4K9N) and GAPDH (3H12) were from Cell Signaling TECHNOLOGY and MBL, respectively. Recombinant hIL-2 (200-02) was obtained from PEPROTECH. RPMI1640 medium deficient in methionine and choline (MCD) was manufactured by Gmep, Inc.

Cell isolation and flow cytometry

To isolate liver immune cells, mice were anesthetized, and liver tissue was perfused with 20 ml warm HBSS ($Ca2^+$ $Mg2^+$ free) via portal vein. Liver was removed, then excised and digested in RPMI1640 supplemented with 0.1 mg/ml DNaseI (10104159001, Roche) and 2 mg/ml Collagenase D

(11088858001, Roche) for 30 min with gentle shaking at 37 °C. After tissue digestion, the cell suspension was passed through a 100-micron cell strainer and centrifuged at 50 X g for 3 min. The supernatant was transferred to a new tube and centrifuged at 500 X g for 5 min. The pellet was resuspended with 17.6 % Optiprep (1893, Serumwerk Bernburg) and overlaid by 8.2 % Optiprep, and then centrifuged at 2000 rpm for 20 min. The cells from interface were obtained and used for flow cytometric analysis. Splenocytes were prepared by mashing through a strainer followed by depletion of red blood cells. Cell suspensions were stained with Fixable Viability Dye eFluor™ 780 (65-0865-18, eBioscience) and anti-CD16/32 (2.4G2, BD Pharmingen) in a PBS buffer supplemented with 0.5% Bovine Serum Albumin (BSA), followed by the indicated antibodies in the dark on ice. For T cell or $CD11b^+$ cell purification, obtained cell suspensions were subjected to magnetic cell separation using $CD4^+$ T Cell Isolation Kit, mouse (130-104-454, Miltenyi Biotec), $CD8a^+$ T Cell Isolation Kit, mouse (130-104-075, Miltenyi Biotec) or CD45 MicroBeads, mouse (130-052-301, Miltenyi Biotec), prior to fluorescent activated cells sorting (FACS). For intracellular staining of Foxp3 and Batf, cell surface marker-stained cells were fixed and permeabilized with Foxp3/Transcription Factor Staining

Buffer Set (00-5523-00, eBioscience) according to manufacturer's instruction. For intracellular cytokine detection, cells were stimulated with 50 ng/ml phorbol 12-myristate 13-acetate (PMA; 79346, Sigma-Aldrich) and 500 ng/ml ionomycin (10634, Sigma-Aldrich) in the presence of GolgiPlug^{TM} Protein Transport Inhibitor (555029, BD Biosciences) for 5 hr. After staining of cell surface markers and fixation, intracellular staining was carried out. For cell death assay, Annexin V staining was performed using Annexin V Apoptosis Detection Kit APC (88-8007-72, eBioscience). All flow cytometry analyses and FACS were performed on BD FACSCanto II (BD) and BD FACSAria III (BD), respectively. Data were analyzed by FlowJo V10 (Tree Star).

RNA extraction and real-time quantitative PCR

Total RNA was isolated from tissue or cells using RNeasy Mini kit (74104, QIAGEN) and cDNA was synthesized with PrimeScript^{TM} Master Mix (RR036A, TaKaRa) according to the manufacture's protocols. Real-time PCR reactions were performed in technical replicates using SsoFast EvaGreen Supermix (172-5203, Bio-Rad) and run with CFX Connect Real-Time PCR system (Bio-Rad). The relative gene expression levels were normalized to the expression of *Actb*. Primers used for quantitative PCR were listed in Supplemental Table 1.

Immunoblot

Liver tissues were homogenized and then lysed for 30 min in a lysis buffer containing 1 % TritonX-100, 20 mM Tris H-Cl (pH 7.4), 150 mM NaCl, 10 mM NaF, 2 mM Na_3VO_4 and cOmplete^{TM} Protease Inhibitor Cocktail (4693116001, Roche). Equal amounts of protein were separated by SDS-PAGE and transferred onto polyvinylidene difluoride membranes. Membranes were blocked with 5% BSA and incubated with the indicated primary antibodies, followed by secondary antibodies conjugated with horseradish peroxidase (anti-rabbit IgG; 111-036-047, anti-mouse IgG; 115-035-003, Jackson ImmunoResearch). Signal was detected with SuperSignal^{TM} West Pico PLUS Chemiluminescent Substrate (32132, Thermo Scientific) and exposed to Xray film (Super RX, FUJIFILM). Densitometry for band quantification was performed by image analysis software, Fiji.

Histological analysis

Liver tissues were collected and fixed with 4 % paraformaldehyde solution and then embedded with OCT compound (4583, Tissue-Tek) or paraffin. The frozen sections were used for Oil Red O staining. To investigate liver fibrosis, the paraffin sections were stained with Sirius red stain or Masson's Trichrome stain. For assessment of liver damage, the paraffin sections were stained with *In Situ* Cell

Death Detection Kit, POD (11684817910, Roche) and stained tissues were visualized by fluorescence microscopy, BZ-X810 (Keyence). For quantification, 5 random microscopic fields in each section were captured, and all images are analyzed by BZ-X Analyzer Hybrid Cell Count and Macro Cell Count software (Keyence).

In vitro suppression assay

Tregs ($CD45^+CD4^+CD25^+Tigit^+$) were sorted from the liver of CD WT and CD dKO mice. Responder naïve CD4 T cells from the spleen of Ly5.1 mice were labelled with CellTrace™ Violet dye (C34557, Invitrogen™) and cultured with sorted Tregs (at a responder-Tregs ratio of 5:1), in the presence of Dynabeads Mouse T-cell activator CD3/CD28 (DB11452, Thermo Fisher) in round bottom 96 well plate for 4 days.

Supplemental Figures

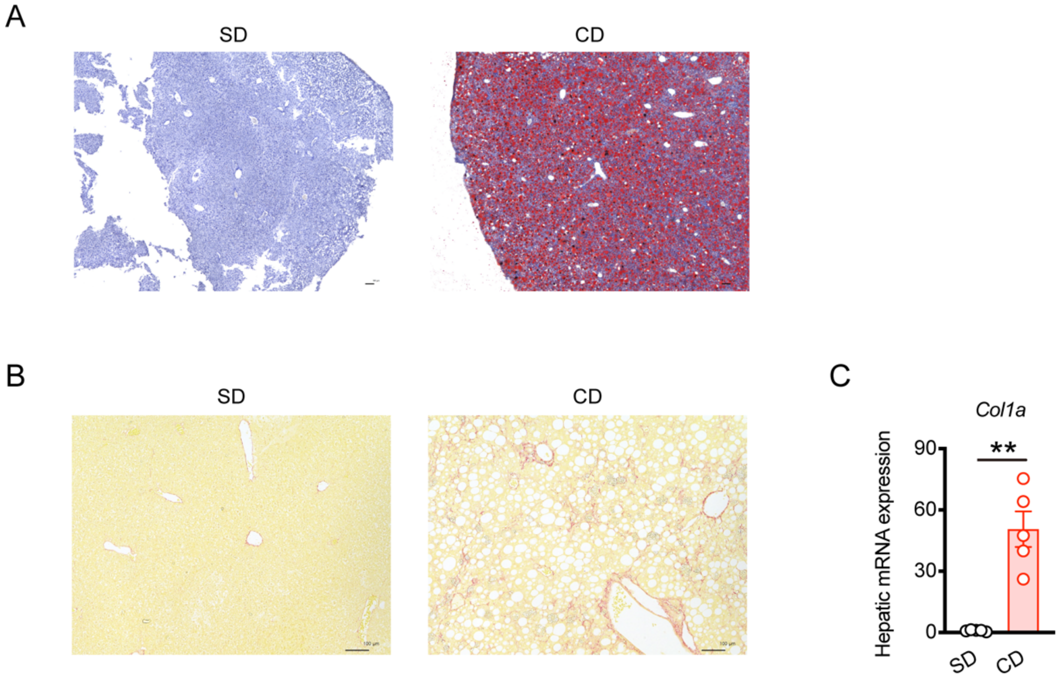

Supplemental Figure 1. Induction of hepatic steatosis and fibrosis in mice fed CD for 8 weeks.

Male C57BL/6 mice were fed SD or CD for 8 weeks. (A) Representative Oil red O staining of liver sections from mice fed SD (left) and CD (right) from two independent experiments. Original magnification 4X, scalebar 100 μm. (B) Representative Sirius red staining of liver sections from mice fed SD (left) and CD (right) from two independent experiments. Original magnification 10X, scalebar 100 μm. (C) mRNA expression of *Col1a* in liver tissue (n=5 per group). Data are means ± SEM. The *p*-value was calculated using unpaired two-tailed student's *t*-test for (C). $^{**}P < 0.01$.

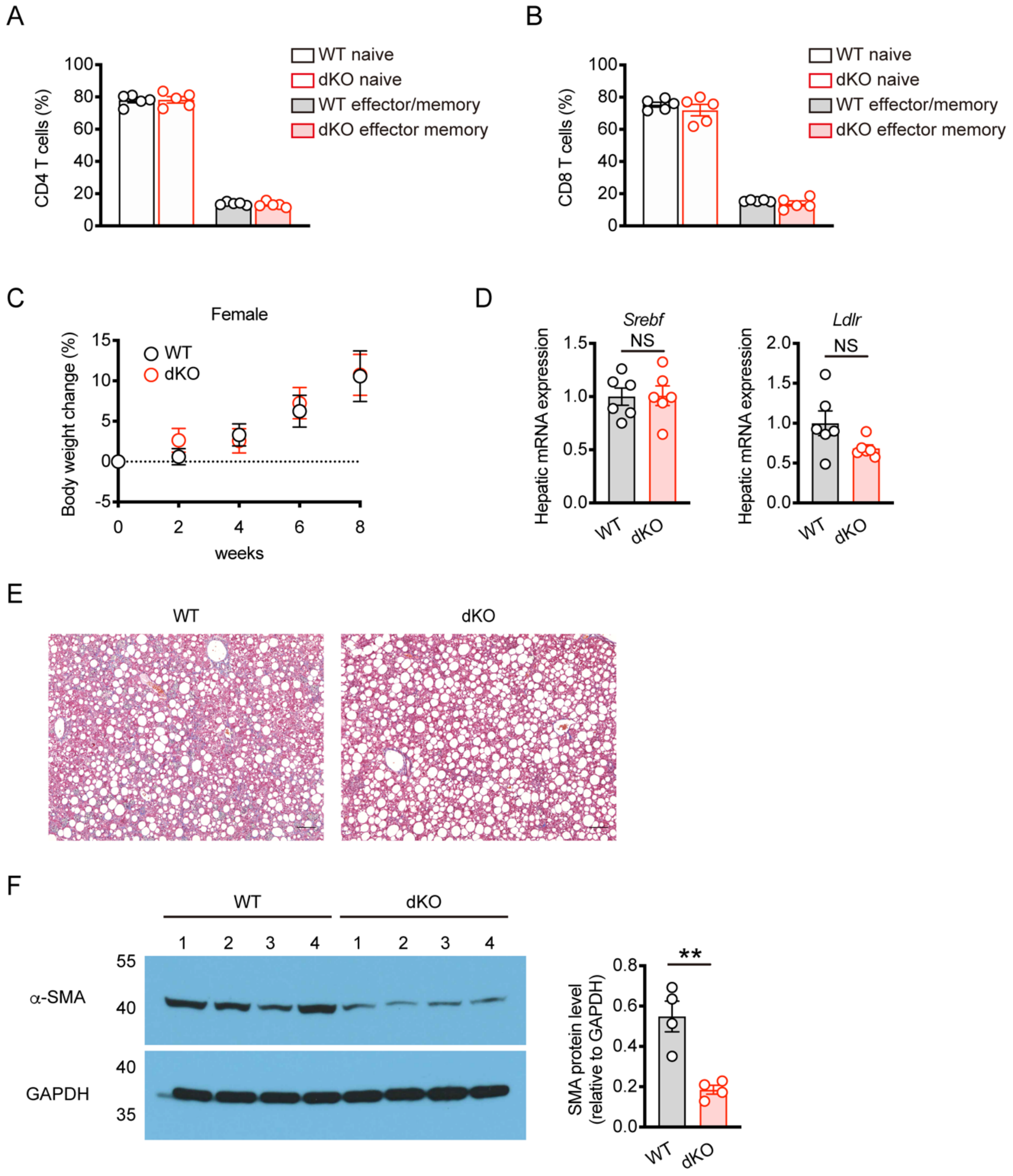

Supplemental Figure 2. Loss of *Nr4a1* and *Nr4a2* in T cell alleviates NASH pathology. (A), (B) Flowcytometric analysis of splenic T cells from WT and dKO mice. (n=5 per group). (A) Percentages of naïve (CD25^{-}CD44^{-}CD62L^{hi}) and effector/memory (CD44^{hi}CD62L^{-}) cells in CD4 T cells. (B) Percentages of naïve (CD25^{-}CD44^{-}CD62L^{hi}) and effector/memory (CD44^{hi}) cells in CD8 T cells. (C) Relative body weight changes of female WT and dKO mice fed CD for the indicated times. (WT; n=9, dKO; n=10). (D)-(F) Male WT and dKO mice were fed CD for 8 weeks. (D) mRNA expression of *Srebf* and *Ldlr* in liver tissue (n=6 per group).

(E) Representative Masson-trichrome staining of liver sections from two independent experiments. Original magnification 10X, scalebar 100 μm. (F) Representative western blotting analysis of α-smooth muscle actin (α-SMA) (left) and quantification of α-SMA protein level relative to GAPDH protein (right) in liver tissue. Each number in the lane represents an individual mouse (n=4 male per group). The *p*-value was calculated using unpaired two-tailed student's *t*-test for (D), (F). **$P < 0.01$, NS no significant difference.

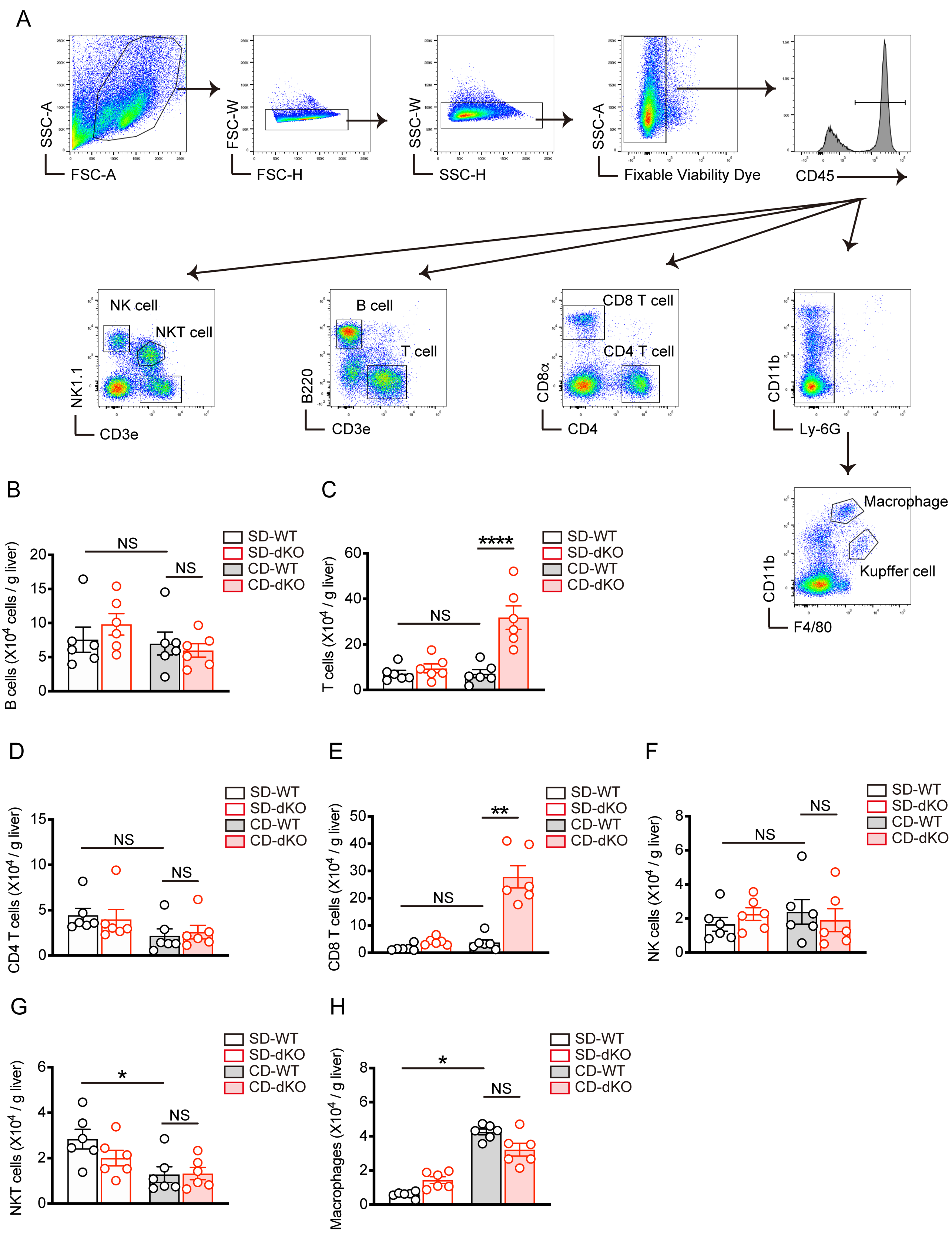
A
SSC-A
FSC-A
FSC-W
FSC-H
SSC-W
SSC-H
SSC-A
Fixable Viability Dye
CD45
NK cell
NKT cell
NK1.1
CD3e
B cell
T cell
B220
CD3e
CD8 T cell
CD4 T cell
CD8α
CD4
CD11b
Ly-6G
Macrophage
Kupffer cell
CD11b
F4/80
B
B cells (X10^4 cells / g liver)
NS
NS
SD-WT
SD-dKO
CD-WT
CD-dKO
C
T cells (X10^4 / g liver)
****
NS
SD-WT
SD-dKO
CD-WT
CD-dKO
D
CD4 T cells (X10^4 / g liver)
NS
NS
SD-WT
SD-dKO
CD-WT
CD-dKO
E
CD8 T cells (X10^4 / g liver)
**
NS
SD-WT
SD-dKO
CD-WT
CD-dKO
F
NK cells (X10^4 / g liver)
NS
NS
SD-WT
SD-dKO
CD-WT
CD-dKO
G
NKT cells (X10^4 / g liver)
*
NS
SD-WT
SD-dKO
CD-WT
CD-dKO
H
Macrophages (X10^4 / g liver)
*
NS
SD-WT
SD-dKO
CD-WT
CD-dKO

Supplemental Figure 3. Immune cell composition in the liver from WT and dKO mice fed SD or CD.

WT and dKO mice were fed SD or CD for 8 weeks (n=6 per group). (A) Flow cytometry of gating strategy for immune cell analysis in the liver of WT mice. (B)-(H) Cell numbers of B cells (B), $CD3e^{+}$ T cells (C), CD4 T cells (D), CD8 T cells (E), NK cells (F), NKT cells (G), macrophages (H) within $CD45^{+}$ cells in the liver. The *p*-value was calculated using One-way analysis of variance (ANOVA) or Kruskal-Wallis test for (B)-(H). *$P < 0.05$, **$P < 0.01$, ****$P < 0.0001$, NS no significant difference.

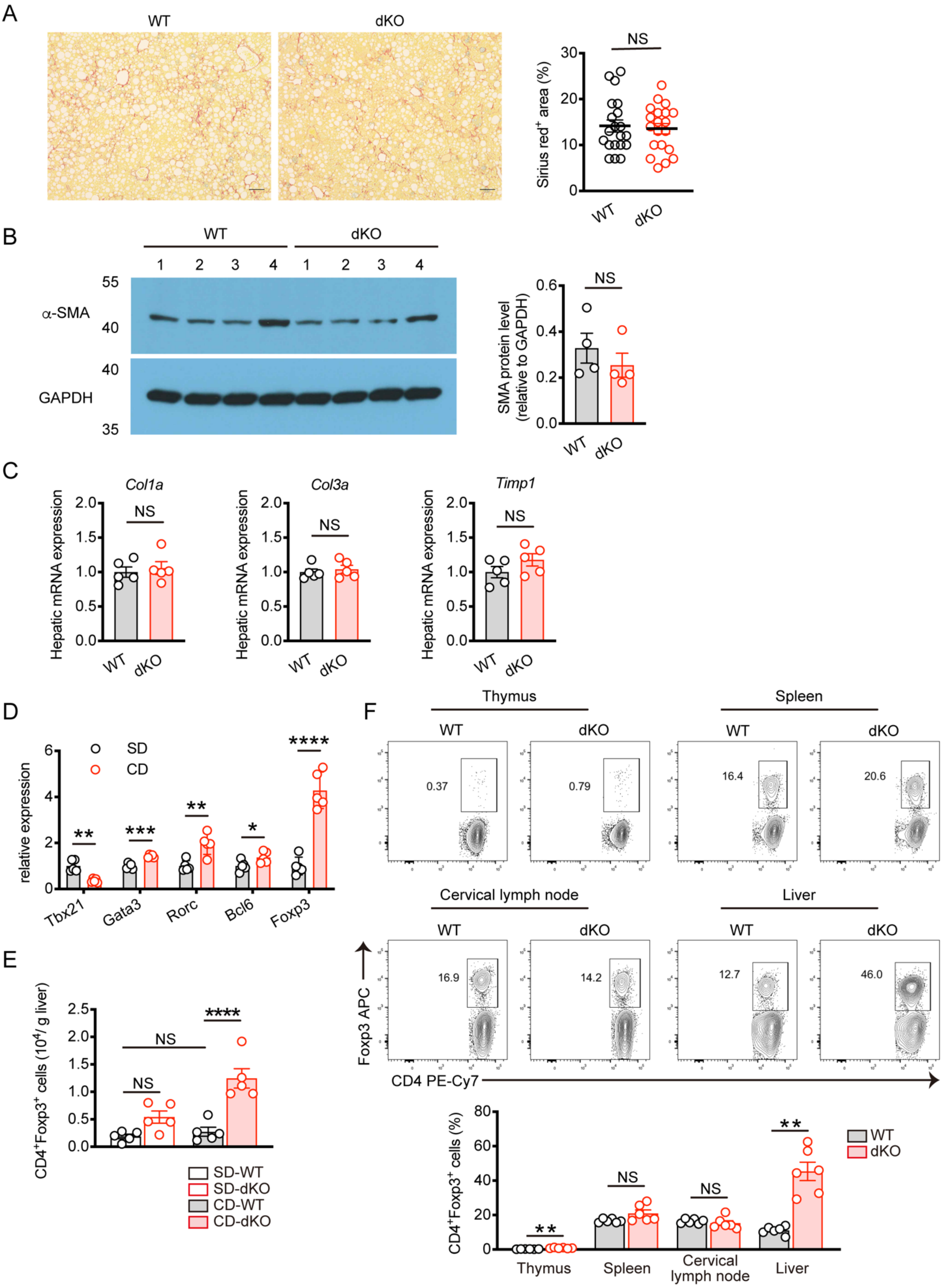
A
WT
dKO
NS
Sirius red+ area (%)
B
WT
dKO
1 2 3 4 1 2 3 4
55
α-SMA
40
40
GAPDH
35
NS
SMA protein level (relative to GAPDH)
C
Col1a
Col3a
Timp1
NS
Hepatic mRNA expression
D
SD
CD
relative expression
**
***
**
*
****
Tbx21
Gata3
Rorc
Bcl6
Foxp3
E
CD4+Foxp3+ cells (10^4/ g liver)
NS
NS
****
SD-WT
SD-dKO
CD-WT
CD-dKO
F
Thymus
Spleen
Cervical lymph node
Liver
0.37
0.79
16.4
20.6
16.9
14.2
12.7
46.0
Foxp3 APC
CD4 PE-Cy7
CD4+Foxp3+ cells (%)
**
NS
NS
**
Thymus
Spleen
Cervical lymph node
Liver

Supplemental Figure 4. Loss of *Nr4a1* and *Nr4a2* in T cells promotes hepatic Tregs accumulation in NASH.

(A)-(C) WT and *Cd8Cre* dKO mice were fed CD for 8 weeks. (A) Representative Sirius red staining of liver sections from male mice. Original magnification 10X, scalebar 100 μm (left). Quantification of Sirius red staining area (%) per field (right) (n=4 male per group). (B) Representative western blotting analysis of α-SMA (left) and quantification of α-SMA protein level relative to GAPDH protein (right) in liver tissue. Each number in the lane represents an individual mouse (1,2,3; female and 4; male). (C) mRNA expression of *Col1a*, *Col3a* and *Timp1* in liver tissue from female mice (n=5 per group). (D) Male C57BL/6 mice were fed SD or CD for 8 weeks. mRNA expression of transcription factors in hepatic CD4 T cells (n=5) Each statistical significance between groups was individually assessed and summarized. (E) Cell numbers of hepatic Tregs from WT and dKO mice fed SD or CD for 8 weeks (n=5 per group). (F) WT and dKO mice were fed CD for 8 weeks. Representative flow cytometry plots (top) and percentages (bottom) of Tregs in the thymus, spleen, cervical lymph node and liver from WT and dKO mice (n=6 per group). continued.

continued

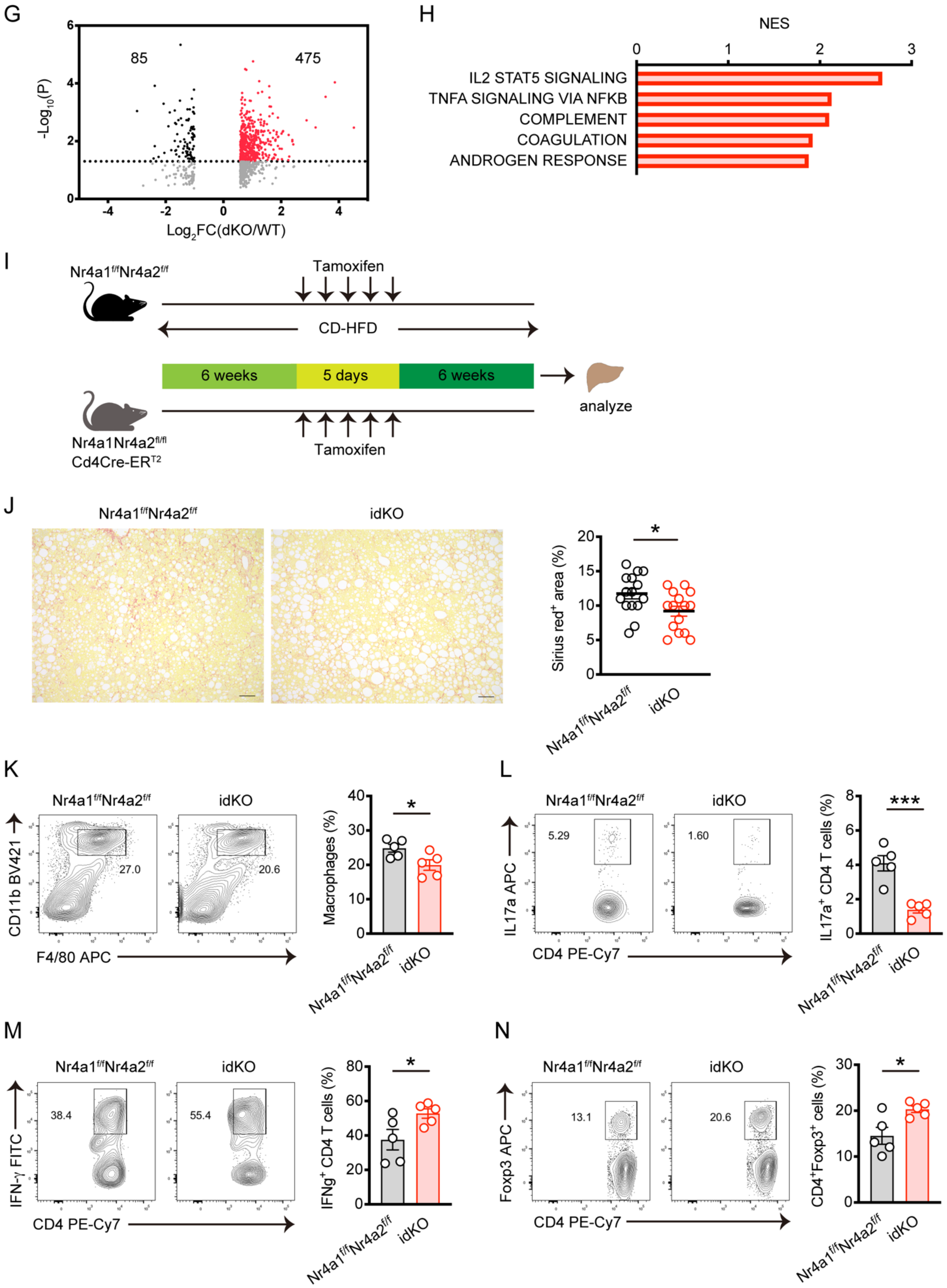

G
85
475
-Log10(P)
Log2FC(dKO/WT)
H
NES
IL2 STAT5 SIGNALING
TNFA SIGNALING VIA NFKB
COMPLEMENT
COAGULATION
ANDROGEN RESPONSE
I
Nr4a1f/fNr4a2f/f
Tamoxifen
CD-HFD
6 weeks
5 days
6 weeks
analyze
Nr4a1Nr4a2fl/fl
Cd4Cre-ERT2
Tamoxifen
J
Nr4a1f/fNr4a2f/f
idKO
Sirius red+ area (%)
K
Nr4a1f/fNr4a2f/f
idKO
CD11b BV421
F4/80 APC
27.0
20.6
Macrophages (%)
L
5.29
1.60
IL17a APC
CD4 PE-Cy7
IL17a+ CD4 T cells (%)
M
38.4
55.4
IFN-γ FITC
IFNg+ CD4 T cells (%)
N
13.1
20.6
Foxp3 APC
CD4+Foxp3+ cells (%)

Supplemental Figure 4. (continued)

(G), (H) WT and dKO mice were fed CD for 8 weeks. (G) Volcano plot comparing the gene expression between WT and dKO hepatic CD4 T cells. Differentially expressed genes are depicted in black and red, respectively. (H) List of significantly upregulated pathway in dKO hepatic CD4 T cells performed by GSEA analysis using Hallmark gene set. NES; the normalized enrichment score. (I) Schematic representation of the experimental procedure of NASH induction mouse model with T cell specific inducible deletion of Nr4a1 and Nr4a2. (J) Representative Sirius red staining of liver sections from male mice fed CD. Original magnification 10X, scalebar 100 μm (left). Quantification of Sirius red staining area (%) per field in NASH-induced WT and idKO mice (right) (n=3 male per group). (K)-(N) Representative flow cytometry plots (left) and percentages (right) of macrophages ($CD45^+CD11b^{hi}F4/80^{int}$) (K), Th17 cells ($CD45^+CD4^+IL17a^+$) (L), Th1 cells ($CD45^+CD4^+IFN\gamma^+$) (M) and Tregs ($CD45^+CD4^+Foxp3^+$) (N) in the liver of NASH-induced WT and idKO mice (n=5 per group). Data are means ± SEM. The *p*-value was calculated using unpaired two-tailed student's *t*-test or Mann-Whitney test for (A)-(D), (F), (J)-(N), One-way analysis of variance (ANOVA) for (E). *$P < 0.05$, **$P < 0.01$, ***$P < 0.001$, ****$P < 0.0001$; NS no significant difference.

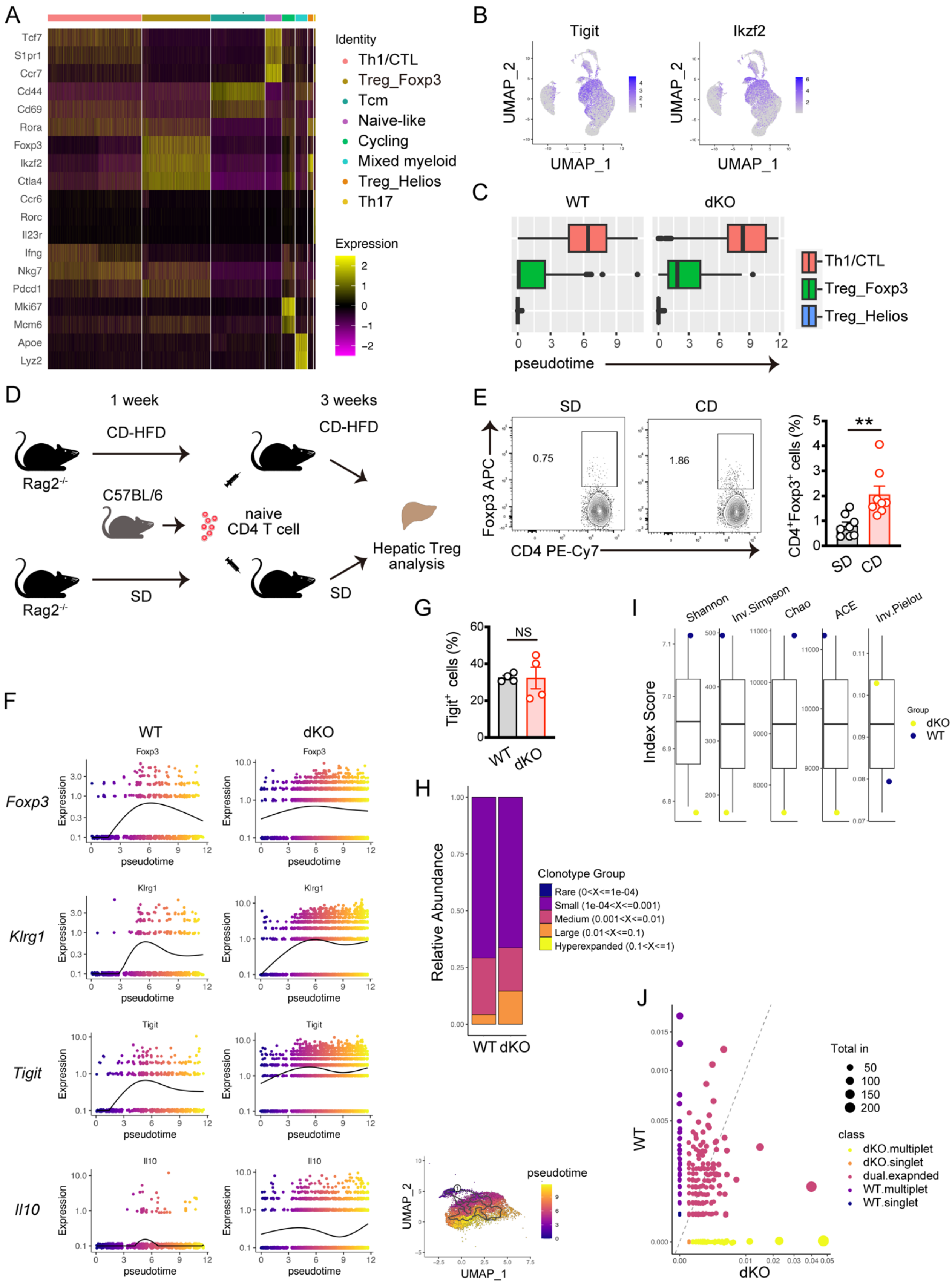
A
B
C
D
E
F
G
H
I
J
Identity
Th1/CTL
Treg_Foxp3
Tcm
Naive-like
Cycling
Mixed myeloid
Treg_Helios
Th17
Expression
Tigit
Ikzf2
UMAP_1
UMAP_2
WT
dKO
pseudotime
1 week
3 weeks
CD-HFD
Rag2-/-
C57BL/6
naive
CD4 T cell
Hepatic Treg
analysis
SD
CD
Foxp3 APC
CD4 PE-Cy7
CD4+Foxp3+ cells (%)
Tigit+ cells (%)
NS
Relative Abundance
Clonotype Group
Rare (0<X<=1e-04)
Small (1e-04<X<=0.001)
Medium (0.001<X<=0.01)
Large (0.01<X<=0.1)
Hyperexpanded (0.1<X<=1)
Index Score
Shannon
Inv.Simpson
Chao
ACE
Inv.Pielou
Group
Total in
class
dKO.multiplet
dKO.singlet
dual.exapnded
WT.multiplet
WT.singlet
Foxp3
Klrg1
Tigit
Il10
Expression

Supplemental Figure 5. Transcriptome analysis reveals clonal expansion of dKO Tregs in the liver during NASH.

(A)-(C) WT and dKO mice were fed CD for 8 weeks. (A) Heatmap of key marker genes in hepatic CD4 T cells. (B) UMAP showing expression of *Tigit* (left) and *Ikzf2* (right) in hepatic CD4 T cells. (C) Cell distribution of Th1/CTL, Treg_Foxp3 and Treg_Helios cluster in pseudotime. (D) Schematic representation of adaptive transfer of WT splenic naïve CD4 T cells into *Rag2*$^{-/-}$ mice fed SD or CD. (E) Representative flow cytometry plots (left) and percentages (right) of Foxp3^{+} cells gated on CD45^{+}CD4^{+} cells in the liver of mice fed SD or CD (n=8 per group). (F)-(J) WT and dKO mice were fed CD for 8 weeks. (F) UMAP presentation of trajectory and pseudotime analysis of hepatic Tregs (Treg_Foxp3 and Treg_Helios cluster). White circle on UMAP represents start point (right). Expression levels of indicated genes in WT and dKO hepatic Tregs clusters over pseudotime (left). (G) Percentages of Tigit^{+} Tregs in the spleen from WT and dKO mice (n=4 per group). (H) Histograms of the relative proportions filled up by clonotypes in hepatic CD4 T cells from WT and dKO mice. (I) Diversity measurement based on clonotypes in WT and dKO hepatic CD4 T cells using Shannon, Inverse Simpson, Chao, abundance-based coverage estimator (ACE) and Inverse Pielou's measure of species evenness indices. (J) Scatter plot showing comparison of clonotypes between WT and dKO hepatic CD4 T cells. The *p*-value was calculated using unpaired two-tailed student's *t*-test for (E), (G). $^{**}P < 0.01$, NS no significant difference.

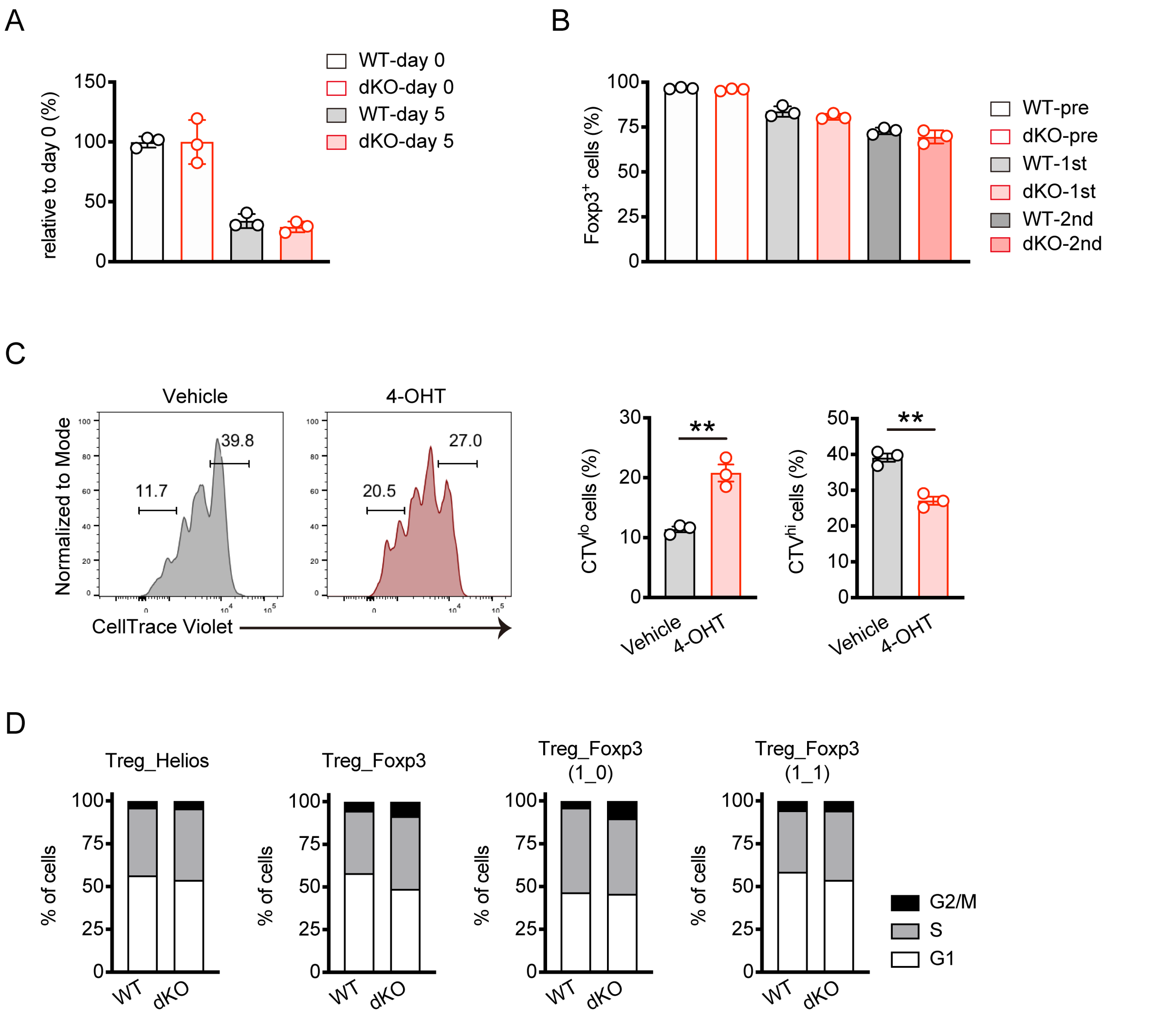

Supplemental Figure 6. Loss of *Nr4a1* and *Nr4a2* in T cells promotes Tregs proliferation. (A) mRNA expression of *Foxp3* in sorted splenic CD4^{+}CD25^{+} Tregs (day 0) and cultured cells for 5 days (day 5) (n=3 per group). (B) Percentages of Foxp3 expressing cells in sorted splenic Tregs and cultured cells. Sorted Tregs (pre) were stimulated for 2 days (1st), rested for 3 days and then restimulated with α-CD3 and α-CD28 antibodies for 2 days (2nd) (n=3 per group). (C) Representative CTV intensity histograms (left) and percentages of CTV^{lo} (middle) and CTV^{hi} (right) cells in splenic Tregs. Sorted Tregs from *Nr4a1*$^{f/f}$*Nr4a2*$^{f/f}$*CreER*^{T2} mice were stimulated with α-CD3 and α-CD28 antibodies in the presence or absence of 500 nM 4-hydroxytamoxifen (4-OHT) for 2 days. Then the cells were rested for 3 days, labelled with CTV and restimulated for 3 days in the absence of 4-OHT (n=3 per group). (D) Cell cycle state was assessed in each Tregs cluster from Figure 5C and 5D using Surat Cell-Scoring for genes indicative of G1, S and G2/M phases. The *p*-value was calculated using unpaired two-tailed student's *t*-test for (C). **P < 0.01.

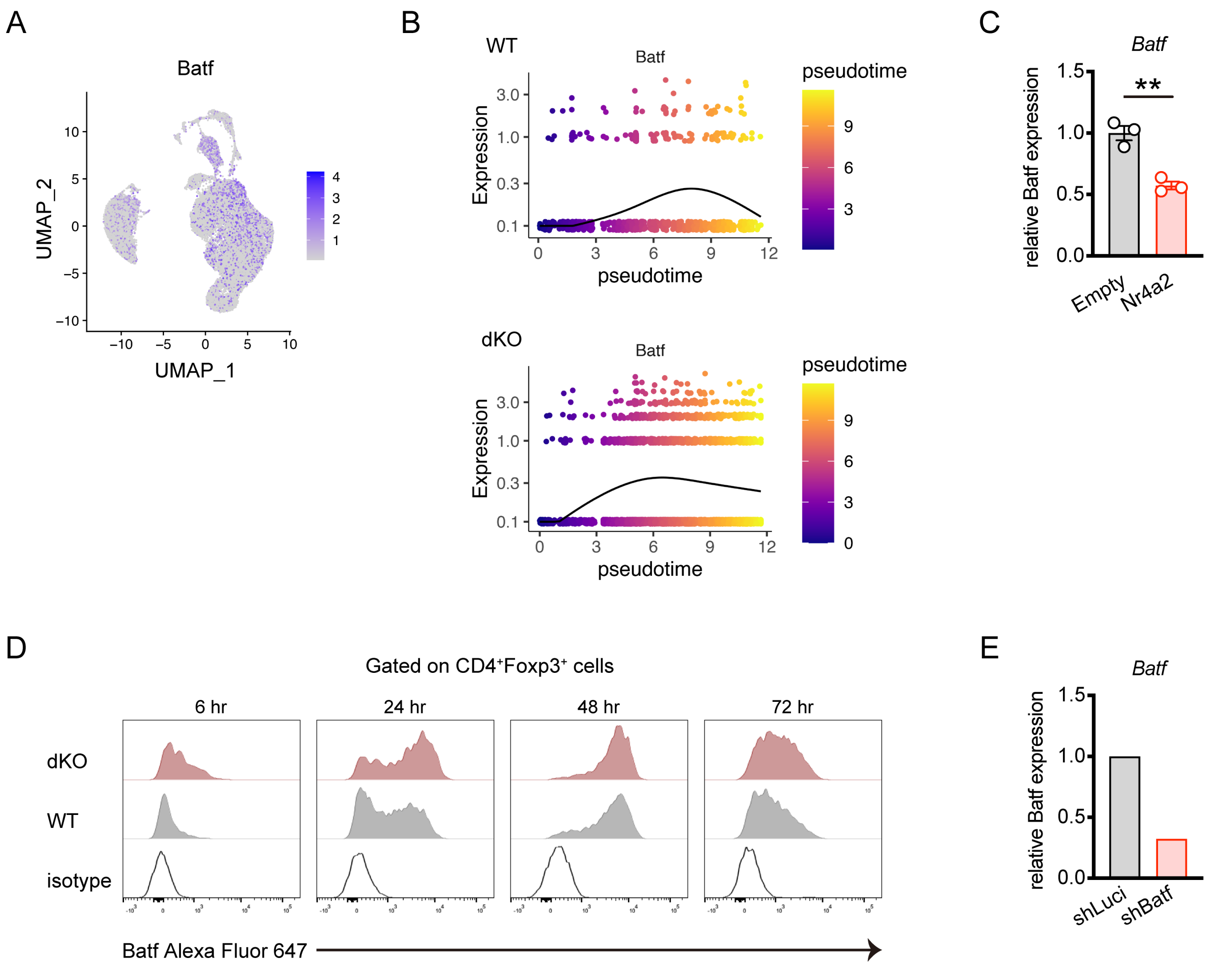

Supplemental Figure 7. Nr4a-Batf axis regulates Tregs proliferation.
(A) UMAP showing *Batf* expression in hepatic CD4 T cells from mice fed CD for 8 weeks. (B) Expression levels of *Batf* in Treg_Foxp3 and Treg_Helios cluster over pseudotime from WT and dKO hepatic CD4 T cells. (C) mRNA expression of *Batf* in splenic CD4 T cells transduced with an empty vector or a vector encoding Nr4a2. Sorted naïve CD4 T cells were retrovirally transduced and rested for 3 days. Then GFP^+CD4^+ cells were sorted and restimulated with α-CD3 and α-CD28 antibodies for 6 hrs (n=3 per group). (D) Representative histograms normalized to mode for Batf expression in WT and dKO $CD4^+Foxp3^+$ T cells from two independent experiments. Sorted CD4 T cells from the spleen were stimulated with plate-bound α-CD3 and α-CD28 antibodies for the indicated times. (E) mRNA expression of *Batf* in splenic CD4 T cells transduced with shRNA targeting *Luciferase* or *Batf*. After resting culture, GFP^+CD4^+ cells were sorted and restimulated with α-CD3 and α-CD28 antibodies for 6 hrs. The *p*-value was calculated using unpaired two-tailed student's *t*-test for (C). $^{**}P < 0.01$.

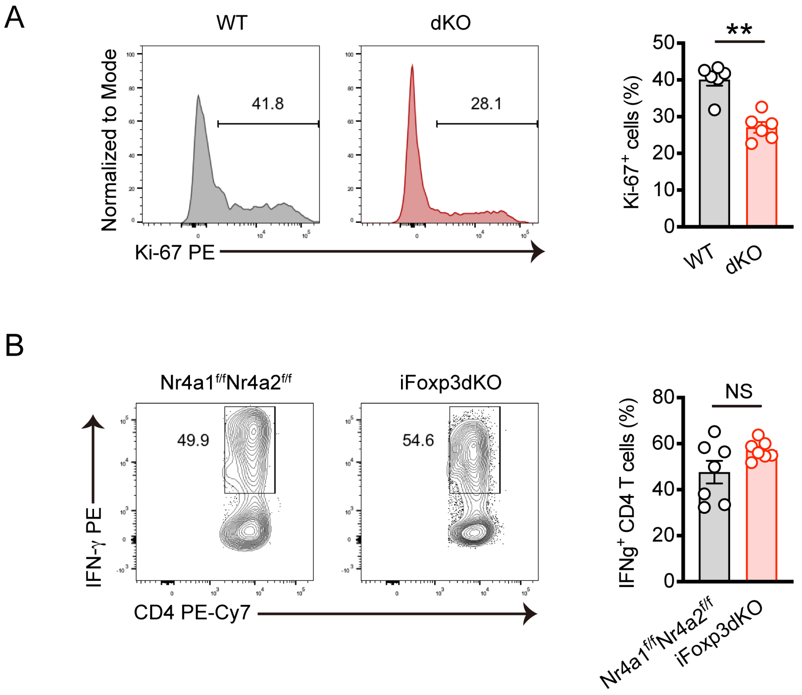

Supplemental Figure 8. The loss of Nr4a1 and Nr4a2 in T cell promotes Tregs function.

(A) Representative histograms normalized to mode for Ki-67 expression (left) and percentages of Ki-67^+ cells (right) within hepatic $CD45^+CD4^+Foxp3^+$ cells from WT and dKO mice fed CD for 8 weeks (n=6 per group). (B) Representative flow cytometry plots (left) and percentages (right) of Th1 cells ($CD45^+CD4^+IFN\gamma^+$) in the liver of NASH induced WT and iFoxp3dKO mice (n=7 per group). The *p*-value was calculated using unpaired two-tailed student's *t*-test for (A), (B). $^{**}P < 0.01$, NS no significant difference.

Supplemental Tables

Supplemental Table 1
Primers for real-time quantitative PCR

Nr4a1-F
5'-TGTGAGGGCTGCAAGGGCTTC-3'
Nr4a1-R
5'-AAGCGGCAGAACTGGCAGCGG-3'
Nr4a2-F
5'-CTGTGCGCTGTTTGCGGTGAC-3'
Nr4a2-R
5'-CGGCGCTTGTCCACTGGGCAG-3'
Nr4a3-F
5'-ACATGTGCCGTGTGCGGCGAC-3'
Nr4a3-R
5'-CGTCTCTTGTCCACTGGGCAG-3'
b-actin-F
5'-ATCCTGGCCTCGCTGTCCAC-3'
b-actin-R
5'-GGGCCGGACTCGTCATAC-3'
Col1a-F
5'-CTGCTGGCAAAGATGGAGA-3'
Col1a-R
5'-ACCAGGAAGACCCTGGAATC-3'
Col3a-F
5'-CAAATGGCATCCCAGGAG-3'
Col3a-R
5'-CATCTCGGCCAGGTTCTC-3'
Timp1-F
5'-CTCAAAGACCTATAGTGCTGGC-3'
Timp1-R
5'-CAAAGTGACGGCTCTGGTAG-3'
Tgfb-F
5'-GAGGTCACCCGCGTGCTA-3'
Tgfb-R
5'-TGTGTGAGATGTCTTTGGTTTTCTC-3'

Spp1-F
5’-CCCGGTGAAAGTGACTGATT-3’
Spp1-R
5’-TTCTTCAGAGGACACAGCATTC-3’
Trem2-F
5’-CTGGAACCGTCACCATCACTC-3’
Trem2-R
5’-CGAAACTCGATGACTCCTCGG-3’
Arg1-F
5’-TGACTGAAGTAGACAAGCTGGGGAT-3’
Arg1-R
5’-CGACATCAAAGCTCAGGTGAATCGG-3’
Nos2-F
5’-GAGACAGGGAAGTCTGAAGCAC-3’
Nos2-R
5’-CCAGCAGTAGTTGCTCCTCTTC-3’
Srebf1-F
5’-CGACTACATCCGCTTCTTGCAG-3’
Srebf1-R
5’-CCTCCATAGACACATCTGTGCC-3’
Foxp3-F
5’-CCTGGTTGTGAGAAGGTCTTCG-3’
Foxp3-R
5’-TGCTCCAGAGACTGCACCACTT-3’
Rorc-F
5’-GTGGAGTTTGCCAAGCGGCTTT-3’
Rorc-R
5’-CCTGCACATTCTGACTAGGACG-3’
Gata3-F
5’-CCTCTGGAGGAGGAACGCTAAT-3’
Gata3-R
5’-GTTTCGGGTCTGGATGCCTTCT-3’
Tbx21-F
5’-CCACCTGTTGTGGTCCAAGTTC-3’
Tbx21-R
5’-CCACAAACATCCTGTAATGGCTTG-3’
Bcl6-F
5’-CACACCCGTCCATCATTGAA-3’

Bcl6-R

5’-TGTCCTCACGGTGCCTTTTT-3’

Batf-F

5’-CTGGCAAACAGGACTCATCTG-3’

Batf-R

5’-GGGTGTCGGCTTTCTGTGTC-3’

Ldlr-F

5’-GAATCTACTGGTCCGACCTGTC-3’

Ldlr-R

5’-CTGTCCAGTAGATGTTGCGGTG-3’

Supplemental Table 2

Batf KD oligonucleotide
5’-
TGCTGTTGACAGTGAGCGAGAGTGAGGACCTGGAGAAACATAGTGAAGCCACAGATGT
ATGTTTCTCCAGGTCCTCACTCTTGCCTACTGCCTCGGA-3’
