# Supplementary material for: The Nr4a family regulates intrahepatic Treg proliferation and liver fibrosis in MASLD models
Source: J Clin Invest. 2024 Oct 15;134(23):e175305. doi: 10.1172/JCI175305 (PMC11601941; doi:10.1172/JCI175305)
Supplement: Unedited blot and gel images [file jci-134-175305-s125.pdf]

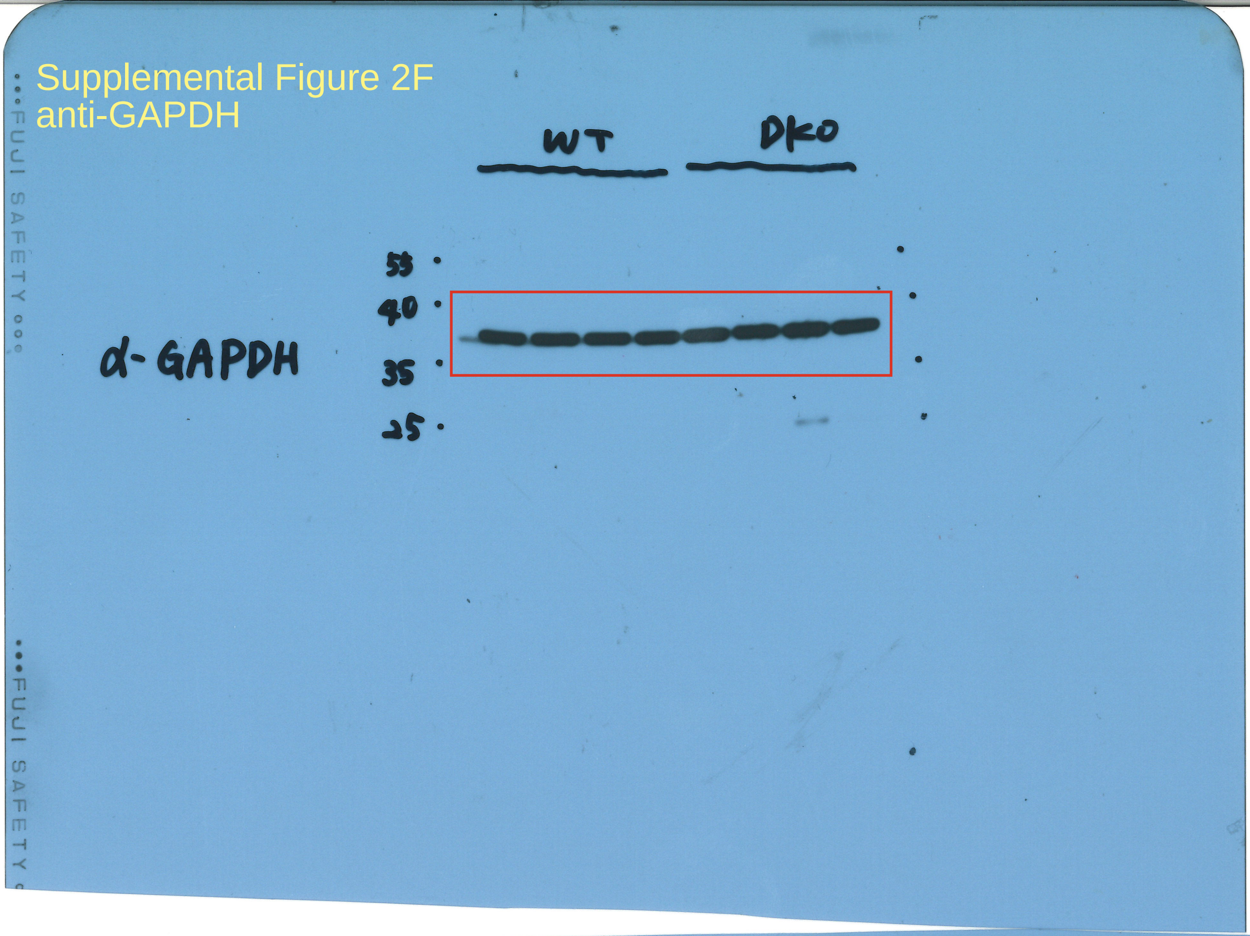
Supplemental Figure 2F
anti-GAPDH
WT
DKO
α-GAPDH
55
40
35
25

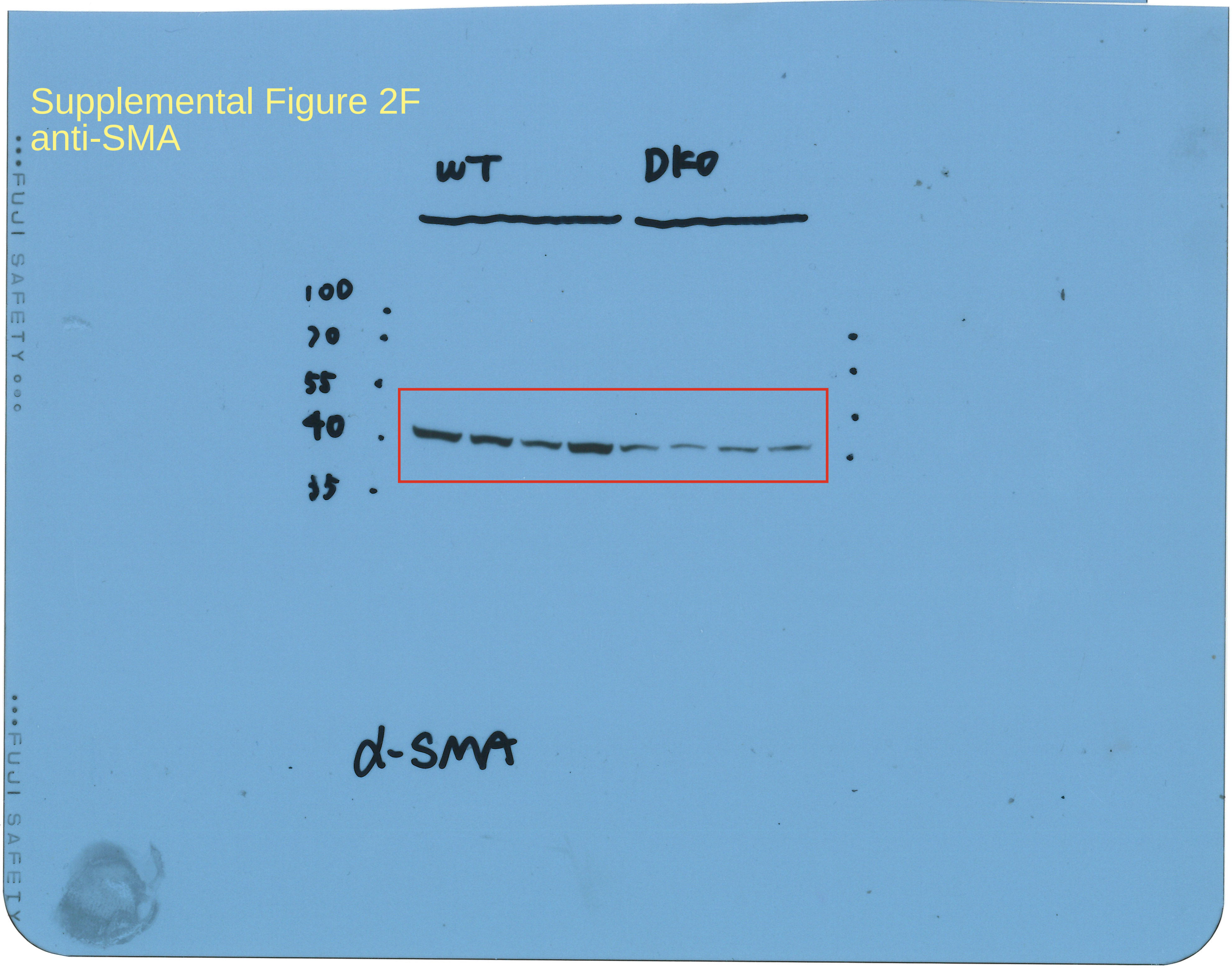
Supplemental Figure 2F
anti-SMA
WT
DKO
100
70
55
40
35
α-SMA

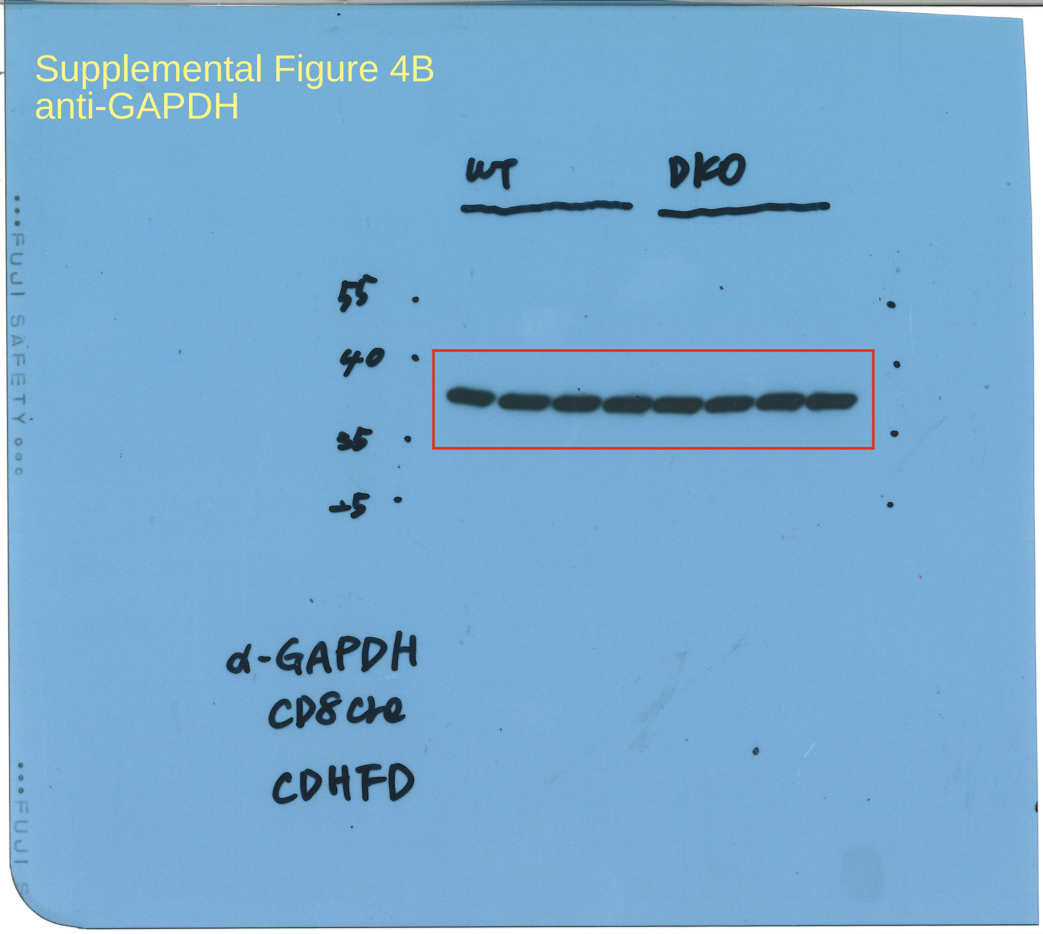
Supplemental Figure 4B
anti-GAPDH
WT
DKO
55
40
35
α-GAPDH
CD8cre
CDHFD

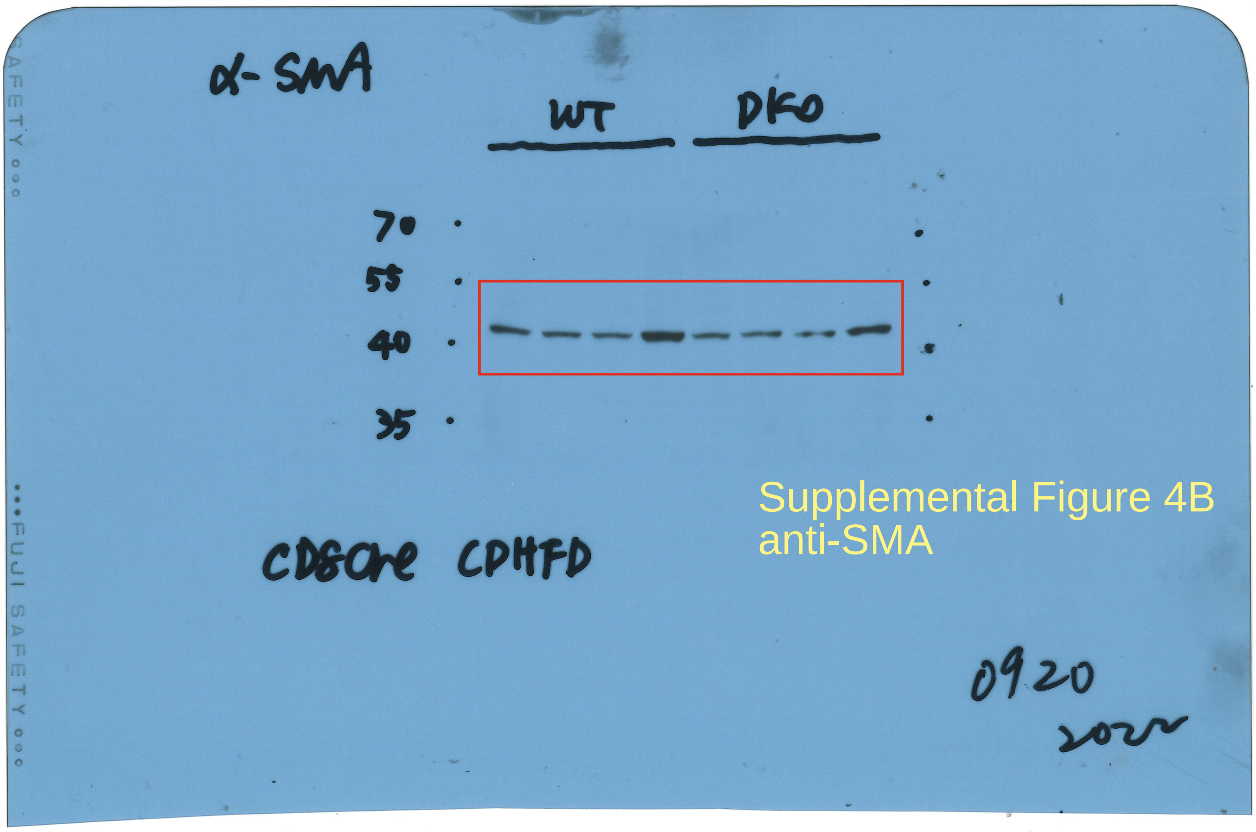
α-SMA
WT
DKO
70
55
40
35
CD8Cre CDHFD
Supplemental Figure 4B
anti-SMA
0920
